# User-Centered Design of Companion Robot Pets Involving Care Home Resident-Robot Interactions and Focus Groups With Residents, Staff, and Family: Qualitative Study

**DOI:** 10.2196/30337

**Published:** 2021-11-01

**Authors:** Hannah Louise Bradwell, Katie Edwards, Deborah Shenton, Rhona Winnington, Serge Thill, Ray B Jones

**Affiliations:** 1 Centre for Health Technology Faculty of Health University of Plymouth Plymouth United Kingdom; 2 Faculty of Arts and Humanities University of Plymouth Plymouth United Kingdom; 3 Auckland University of Technology Auckland New Zealand; 4 Donders Institute for Brain, Cognition and Behaviour Radboud University Nijmegen Netherlands

**Keywords:** companion robots, social robots, Paro, older adults, dementia, care homes, engagement, acceptability, gerontology, Joy for All, social care, user-centered design

## Abstract

**Background:**

Globally, pressure is increasing on health and social care resources due to the aging population and growing prevalence of dementia. Companion robots, such as Paro, demonstrate strong potential for helping reduce this pressure through reported benefits including reduced agitation, depression, loneliness, care provider burden, and medication use. However, we previously identified that user-centered design of robot pets is both essential and understudied. We observed that commonly used robot pets are poorly matched to end-user requirements, and that end users and developers of robot pets differ significantly in their perception of appropriate design. This may explain some of the contradictory outcome research and variance in results for robot pets, such as Paro.

**Objective:**

In response to the literature gap, we aimed to provide user-centered insights into the design of robot pets from key stakeholders to inform future robot development and the choice of robots for real-world implementation and research. We focused on understanding user requirements.

**Methods:**

We conducted a qualitative study with 65 participants from 5 care homes (26 care home residents, 29 staff members. and 10 family members). Care home residents formed groups of between 3 and 4 individuals and experienced free interactions with a range of 8 companion robots and toys, including Paro and more affordable alternatives. The robots provided had a range of esthetics, shell types, interactivity levels, and designs for comparison. Care staff and family members observed the interactions. All participants then engaged in focus groups within their stakeholder category to discuss preferences and user requirements in companion robot design. Both free interactions and focus groups were video and audio recorded, transcribed, and subjected to thematic analysis.

**Results:**

Care home residents, family members, and staff were open and accepting of the use of companion robot pets, with the majority suggesting that they would keep a device for themselves or the residents. The most preferred device was the Joy for All cat, followed by the Joy for All dog. In discussions, the preferred design features included familiar animal embodiment (domestic pet), soft fur, interactivity, big appealing eyes, simulated breathing, and movements. Unfamiliar devices were more often seen as toy-like and suitable for children, producing some negative responses.

**Conclusions:**

This work provides important and user-centered insights into future robot designs for care home residents by means of a comprehensive comparison with key stakeholders. This work strongly supports the use of familiar embodiment in future robot pet designs, with domestic cat and dog morphologies appearing most acceptable. The results have implications for future robot designs and the selection of robot pets for both research and real-world implementations.

## Introduction

### Background

The population worldwide is undergoing a demographic shift, and with life expectancy increasing, a greater proportion of the population is of retirement age or above [1]. This puts pressure on health and social care resources [2], because human function generally deteriorates with age [3]. Due to a lack of resources, there is increasing reliance on pharmacology in care homes [4], which can be problematic due to serious side effects, increased risk of cardiovascular events [5], and mortality [6]. Steptoe et al [7] suggested that these challenges indicate an increased need for research on maintaining well-being. One psychosocial method of improving well-being is the use of companion robots [8]. The most researched companion robot is Paro the seal [9-11], with reported benefits including reduced agitation and depression in dementia [12,13], more adaptive stress response, reduced care provider burden [14], and significantly improved affect (feelings/emotions) and communication between dementia patients and day care staff [15]. Further research has suggested that Paro may reduce psychoactive and analgesic medication use [16], and even decrease blood pressure [17]. However, a particular challenge with wider implementation of Paro is its price of approximately £5000 (approximately US $6900), which limits the number of people able to benefit [18]. Care staff in previous work suggested that this price is too high for care homes [18], demonstrating that the device is poorly matched to the context of use.

Furthermore, the positive results have been questioned as being overly optimistic [19]. A comparison between an active Paro robot and a plush toy found that the benefits of the Paro robot were limited to only engagement [8]. Robinson et al [20] found no main effect for depression (seeing a significant decrease for only loneliness). Thodberg et al [21] compared live dog visits to Paro sessions over 6 weeks and found no improvement in depression with either approach. Moyle et al [22] also found considerable variation in responses to Paro in a large randomized controlled trial. The variation may have resulted from many factors, such as participant loneliness and therefore the need for such devices, and participant like/dislike of animals. However, it is possible that design flaws limit more wide-spread acceptance. For example, research assessing the suitability of Paro for a dementia unit suggested that it may need adapting for such settings as, for instance, its vocalizations can be distressing [23]. Furthermore, while robot pet comparisons have been lacking [9], older adults expressed a significant design preference for pets with familiar embodiments (cats and dogs) when alternatives were provided for comparison with Paro, which demonstrated poor acceptability among older people when preferred devices were available [24]. It is therefore possible that the design of Paro does not match user requirements, in addition to the poor matching of the user context in terms of affordability for real-world adoption. Robot pet implementation and impact may be more consistent with a user-centered design approach to ensure devices match user requirements and the context of use.

User-centered design is the process of involving stakeholders in all stages of product development to create products that are effective, efficient, and satisfactory for the goals of the specific user [25]. Moyle et al [2] suggested involving consumers in conceptualization, development, and testing of companion robots as this may improve appropriateness and practicality to promote acceptability and thus ultimately usage [26]. Daly-Jones et al [25] proposed a cycle of the following 4 key activities: specify user/organizational requirements, understand and specify the context of use for the device, produce prototypes, and conduct user-based assessment. This study therefore aimed to address the first of these activities and provide the understanding and specification of user requirements by engaging key stakeholders in robot evaluations and design discussions.

The design and cost challenges of Paro are problematic considering the large selection bias toward Paro in companion robot research [9-11], thus limiting formation of an evidence base for alternative devices and restricting the understanding of end-user perceptions. Our previous work [24] identified the importance of user-centered design within this field by comparing perceptions of older adults (as end-users) and roboticists (as developers) toward suitable design for a robot pet for older adults. Our results demonstrated significant mismatch in perceptions, with older adults preferring familiar and less sophisticated devices, such as the Joy for All (JfA) cat and dog, and roboticists favoring the potential of Paro. However, we had a relatively small sample of older participants who were more independent than care home residents and were living instead in supported living settings. Therefore, this study aimed to provide insights on user requirements for care home residents to inform user-centered design of companion robots, with implications for future robot design.

### Previous Research

Kachouie et al [9] conducted a review and noted the lack of available companion robot comparison studies, which limits the ability to compare Paro with alternatives and understand user-centered design requirements. The few available comparison studies include the work of Heerink et al [27] who compared 4 robots and asked care providers which features were most important. Additionally, 15 people with dementia interacted with each robot for 1 minute, with researchers observing and counting reactions, such as hugs, kisses, and smiles. The results from care providers suggested that the most important features were having soft fur, looking like a real-life pet, and having appropriate sounds, among others. An issue with this research, however, is the primary focus on care provider perceptions, rather than the opinions of older adults themselves as end-users of the devices. Research has suggested that a person’s stakeholder category can influence technology acceptance [28,29]. Perceived requirements for support in health care can vary among various stakeholder groups, from patients to informal caregivers to professionals [30], and therefore preferred features may differ between the categories of end-users and care providers. The research also failed to include Paro for comparison. As Paro is the most well-researched companion robot available [10], it appears essential for any comparison of companion robots to include Paro. In response, we compared alternatives to Paro directly. A further possible limitation of the study by Heerink et al [27] is the apparent lack of randomization of robot presentation order, which may have introduced bias, as well as reliance on observation. Weaknesses of observational approaches include the Hawthorne effect, observer bias, missed information during live observation, and limited means of validating observed events after observation [31]. In response, we used recording equipment to allow multiple researchers to review and analyze the results, as done in previous research with Paro and older adults [8], resulting in improved validity.

Lazar et al [32] likewise aimed to “rethink” the design of robot pets for older adults and conducted focus groups with 41 independently living older adults, with discussions on issues around companion robots, such as the fiction of a robotic animal, the social role of the robot, and reciprocity. Participants were introduced to 6 devices. The results suggested that some tension existed toward robots as companions, particularly with reference to fiction and lack of human contact. Participants preferred soft, cuddly, and entertaining devices. An issue with this research however was that none of the 6 devices included were designed for older adults specifically, and they were primarily brightly colored children’s toys. Using robots in contexts for which they are not designed can perpetrate negative stereotypes [2], potentially explaining the frictions noted from older adults toward the use of such devices.

Previous research has similarly investigated the use of different esthetics and behaviors of robots. Jones et al [33] provided robots with varying degrees of zoomorphic dog-like behaviors to general population participants and explored, using Likert scales, satisfaction and the willingness to persevere in the interaction. They found that neither look nor behavior impacted participant ratings of performance, and that there was no significant difference in self-reported frustration, excitement, or persistence with the interaction. This could suggest that zoomorphic design is unnecessary. However, it is possible that since the 2008 study, advances in robotics have improved the mimicking of animal behavior. Furthermore, a potential issue with the research is the use of the Roomba robotic vacuum cleaner. Despite being decorated with eyes, ears, a tail, and spotty fur, this robot was not specifically designed as a companion, which perhaps limited participant ability to relate and respond to the robot in either a zoomorphic or nonzoomorphic condition. In response, we compared a range of robot esthetics and behaviors, including animal robots and toys designed as companions, with some specifically for older adults.

The available literature demonstrates limitations in prior work, including a lack of appropriate devices for comparison [27,32,33] and focus only on a single device, limiting informed opinions on features and design [8,34,35]. Previous work has also noted that much robot design research has focused on only 1 stakeholder group [28], such as care staff [27] or independent older adults [32], and that users’ needs and experiences in relation to robot pets remain unexplored [36]. Here, we aimed to help address this situation.

## Methods

### Design

**Figure 1:**
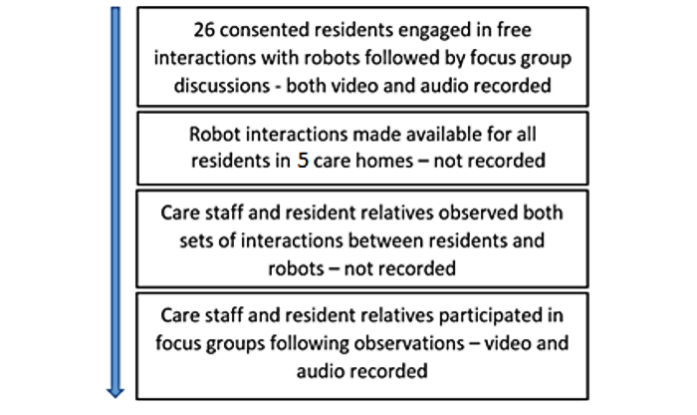
Data collection flowchart.

This was a qualitative user-centered design study in 5 care homes involving free interactions and focus groups. Care home residents consented to participate and engaged in free interactions with devices, followed by focus groups, both of which were recorded. Interactions with the devices were then allowed for all other residents in the home wishing to experience the pets, for equity and practicality, although these interactions were not recorded. Staff and residents’ relatives observed both sets of resident-robot interactions before completing separate focus groups (Figure 1). Ethical approval was given by the University of Plymouth Faculty of Health ethics committee. All participants taking part in the focus groups had the mental capacity to give consent to take part in the research.

### Materials

General materials included 2 video cameras and note pads. The video cameras were used to capture audio recordings for transcription and analysis, and the video also ensured that researchers analyzing transcripts could check which robot residents were referring to, but the footage was not otherwise used for analysis. The use of recording equipment allowed greater validity than observational methods used previously [27]. Video and audio recordings are suggested to provide greater ecological validity, and the data more accurately reflect the experience and environment under analysis than traditional observational notes [37]. Furthermore, with this approach, recordings can be reviewed after the event to validate observations, missed information can be reduced, and analysis can be conducted by multiple researchers, limiting observer bias and improving the overall quality of the analysis [31].

#### Robots

**Figure 2:**
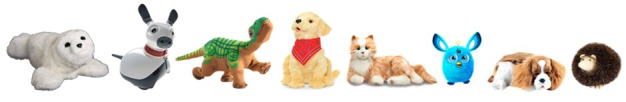
Robots used in the study. From left to right: Paro, Miro, Pleo rb, Joy for All dog, Joy for All cat, Furby, Perfect Petzzz dog, and Hedgehog.

This research used 8 robots and toys for comparison as displayed in Figure 2. The robots selected provided a range of familiar or unfamiliar/mythical embodiments, a variety of soft furry or plastic shells, and varied interactivity types and technological sophistication. Familiar robots were represented by domestic pets (cats or dogs), being animals the general population is more familiar with, whereas animals not commonly found as domestic pets were considered unfamiliar.

The devices included provide a range of esthetic, technological, and behavioral features for comparison. Some (Paro, Miro, and Pleo) are undisputed robots, with technological sophistication allowing for intelligent responses. Most provide vocalizations, interactivity, and movements (Paro, Miro, Pleo, JfA cat and dog, and Furby), while some are passive or inert (Perfect Petzzz [PP] dog and Hedgehog).

### Procedure

Researchers (HB, KE, and DS) visited 5 care homes and set up robot interaction stations in spare rooms, with a table and chairs for participants to be seated. Residents, staff, and family members were informed about the study ahead of the visit, and were invited to attend and participate. All residents with the ability to consent were invited to take part if they desired. Residents, staff, and family members provided written informed consent for both recorded robot interactions and focus groups. Residents were invited into the room in groups of 2 to 4, with staff and family members invited to observe resident interactions without interference in the session. During a session, robots were presented in sets, with Pleo, Miro, and PP dog in one set; Paro, JfA dog, and JfA cat in another set; and Furby and Hedgehog in the last set [24]. The order of set presentation was randomized with a random number generator for each group of participants to avoid presentation bias.

Residents engaged in interactions with the devices during the initial demonstrations, with each set of devices presented for around 10 to 15 minutes. After approximately 30 to 45 minutes of interactions with the 3 sets, researchers brought all devices back onto the table and commenced the focus group discussions. Nygård [38] mentioned that the use of reminders can aid in data collection for those with declining memories; thus, visibility of all devices was important during discussions. We adopted a structured interview schedule (Textbox 1), which was used for all stakeholder categories, with family members and staff being asked to consider care residents in their responses. The staff and family members were asked additional questions around practicalities of implementation, which are not reported here. The care staff and residents’ relatives combined took part in separate focus groups following observation of resident interactions, in order to allow for informed opinions. The staff and family members observed not only the interaction sessions of the consenting residents, but also the free interactions among all care home residents facilitated following completion of the consenting resident focus groups (these whole-home free interaction sessions were not recorded). This ensured that all residents were provided with the opportunity to experience the robots, and allowed the staff and family members to provide informed opinions even when a small number of residents had consented to the focus groups. The duration of the focus groups was 30 to 60 minutes.

**Focus group questions.**

1. Preference?
2. Reason for preference?
3. Thoughts on a new robot design?
4. What should a robot pet be able to do?
5. How should it feel?
6. What expressions and behaviors should it demonstrate?
7. What features or designs should we avoid?
8. Should it be capable of talking and human speech?
9. Should robot pets be personalizable? Should residents be able to pick their design or even be involved in creating their robot, such as knitting, crocheting, or selecting animal/color/fabric?
10. Would a realistic or unrealistic design be the best?
11. If we could leave one of the devices here today, would you want one kept? If so, which one?

### Data Analysis

Audio recordings of the resident-robot free interactions and focus groups were transcribed verbatim and analyzed using NVivo software (QSR International) and deductive thematic analysis. Thematic analysis is a useful and flexible method to generate a rich yet detailed and complex account of qualitative data [39]. Deductive analysis was selected as the research explored perceptions in relation to specific questions. Common threads were identified across all available data, through familiarization, initial code forming, and collating codes into themes before checking the themes, defining them, and reporting them here. Analysis was conducted by 2 researchers (HB and KE), with initial codes compared and subsequent themes coproduced. Coding was reflexive and evolved throughout the analysis, with initial codes being split, combined, or renamed as researchers developed conceptualization of the data [39]. The agreement of 2 researchers aids in the validity of a compelling interpretation. Free interactions of the residents have been reported entirely thematically, while focus group results have been displayed somewhat numerically alongside qualitative quotes, due to answers pertaining to specific questions (structured interview schedule) suitable for numerical comparison, based on the codes and counts of evidence.

## Results

### Participants

Five care homes participated, and from these, we recruited 65 participants (Table 1) comprising 3 sets of stakeholders perceived as influential in companion robot implementation, including residents, staff, and family members. The 5 care homes comprised a purposive sample where the manager was willing to participate, but included a range of residents from those more able to those requiring significant levels of support. Home 1 cared for people with physical disabilities and frailties, and those requiring personal care and support with activities of daily living (ADLs). Most residents in home 2 were quite able and could perform their own ADLs. Home 3 included many residents who had dementia of varying stages and required support with ADLs. Home 4 was a nursing home with residents who were more dependent, and many had dementia, mental health conditions, hearing impairments, stroke, and physical disabilities, and were quite immobile and reliant on support for ADLs. Finally, home 5 had residents who were generally quite able, with few having dementia (although some had signs of confusion); thus, they did not require much care. Four of the homes were residential care homes, while one was a nursing home, differing in the provision of care by a registered nurse. The majority of participants were women (Table 2). While all residents were invited to interact with the robots and devices, the inclusion criteria for video-recorded interactions and focus groups were as follows: capacity to provide informed consent and willingness to participate. Any resident without the capacity to consent was excluded from direct data collection.

The following results are presented in 2 parts: (1) themes generated during thematic analysis of care home residents’ free interactions with the devices, providing insights into the design and feature perceptions of currently available devices, and (2) focus group results with residents, staff, and family members discussing the design of a new companion robot.

**Table 1:** Participants and care homes.

Care home number	Care home description				Participants				Focus groups	
	Beds	Type	Age range of residents (years)	Residents (N=26)		Staff (N=29)	Family members (N=10)	Residents		Staff and family members
1	20	Residential	80-100	8		2	3	2		2
2	14	Residential	75-103	2		3	2	1		1
3	46	Residential	80-100	6		2	0	2		1
4	37	Nursing	70-98	2		12	4	1		5
5	26	Residential	62-107	8		10	1	2		2

**Table 2:** Distribution of participants by gender and stakeholder group.

Gender	Residents (N=26)	Staff (N=29)	Family members (N=10)	Total (N=65)
Male	6	1	2	9
Female	20	28	8	56

### Section 1: Thematic Analysis of Free Interactions

#### Themes During Free Interactions

During the free interactions residents engaged in prior to the focus group discussions, analysis identified 5 key themes, namely, familiarity of design, robot actions, embodiment, acceptability, and robots as a focal point. While some evidence is presented in the narrative below, further example evidence is available in Multimedia Appendix 1. Each quote is provided with a unique identifier, with P representing participating resident, followed by the care home number.

##### Familiarity

Evidence during the free interactions strongly supported a preference for familiar embodiment through codes involving (1) preference for a familiar animal, (2) plastic and unfamiliar devices as infantilizing, (3) unfamiliar devices as unrecognizable, and (4) robot rejection. Residents repeatedly expressed a preference for “something that looks like an animal” [P5_Home_5], stating

I prefer more natural things, the best one is that cat

P1_Home_4

This would suggest a preference for animal embodiment based on domestic pets that older people are likely to have experience with and be familiar with. The unfamiliar devices were described as “not the sort of creature you’d find in a home [Paro],” although despite the incongruent embodiment, Paro was “still my favorite because it’s so soft” [P3_Home_5]. In contrast, another resident felt unable to enjoy Paro and stated

You live in the water and I hate the sea [Paro]

P4_Home_5

Another resident stated they disliked Paro “because it’s not natural” [P7_Home_3], perhaps referring to having a seal in the home or to petting a seal on their lap. Unfamiliar Pleo was even told

Well, nobody could love you like your mother could they, no no no, I’m sorry

P1_Home_5

Further to generally being less preferred, unfamiliar devices were seen as more suited to “children” [P1_Home_5]. Other comments were as follows:

Popular with young children [Miro]

P2_Home_4

Younger child would like to play with these [Miro]

P2_Home_4

That’s alright for children [Pleo]

P5_Home_1

My great granddaughter would love that [Pleo]

P11_Home_1

A tiny little boy might like [Miro]

P11_Home_1

I should give a child something like this [Furby]

P6_Home_1

More appropriate for young children, they’d love this [Paro]

P2_Home_5

It is possible therefore that the toy likeness of devices could create feelings of infantilization. Such comments were almost entirely made toward either Paro, Pleo, Furby, or Miro. Some residents even stated “we must be crazy” [P7_Home_5] and “we’re nuts, we’re nuts” [P5_Home_3], when interacting with Pleo and Paro, although 1 resident interacting with Paro and JfA dog also felt “people will think I’m stupid if they see me now” [P1_Home_2]. While participants were happy and jovial, some clearly felt that some robot designs were unsuitable.

You’re making fools out of us, do you know that? [Paro]

P4_Home_5

They stated that it appeared toy-like, and unfamiliar designs created the least positive responses. Participants sometimes reported unfamiliar devices as unrecognizable, suggesting that the hedgehog “could be a duck” [P1_Home_3] or “a baaa lamb” [P1_Home_3], and that Furby may be “a bat” [P1_Home_4]. There were however several accounts of robot rejection.

[Shown pleo] [bats it away] not for me

P4_Home_3

I don’t want it [Furby]

P4_Home_3

These were all related to Pleo, Furby, Miro, the hedgehog, or Paro.

The white one I wouldn’t go for. I don’t know. She’s a bit, no, there’s nothing to encourage me to touch it. No I couldn’t do it. No I would go away from it [Paro]

P5_Home_3

The unfamiliar Paro also triggered surprising schemas, with 1 resident suggesting they would eat the seal “for tea tomorrow night” [P1_Home_1], and another 2 residents commenting on how people “skin you to make a coat” [P4_Home_5] or how they are “skinned alive when they are born” [P5_Home_1]. Use of familiar embodiment thus seems important for older adults to enhance positive response and recognizability, and to reduce infantilization and chances of rejection.

##### Robot Actions

Residents certainly supported the importance of movement and interactivity in devices, through the code *Important Expressions and Behavior*. On interacting with the JfA cat, 1 resident commented “I like him […] because of his activity and response” [P5_Home_5], and another commented “the cat is very good isn’t it, active” [P3_Home_5]. The residents seemed to understand that most robots were interacting with them.

When you talk, it will answer. When you talk it will answer, because it can hear the vibrations from your voice. That's why she answers

P5_Home_1

Participants particularly praised the dog “moving his face” [P3_Home_3] and the cat “purring” [P5_Home_1]. They also praised devices blinking their eyes.

Oh look at the eyes closing [Paro]

P1_Home_4

The eye blinking is lovely [Cat]

P2_Home_4

The eyes of the devices appeared important, with Furby’s eyes described as “nice animated eyes, that’s really special” [P3_Home_5]. Noninteractive devices were viewed as “just an ornament really, I like the movement ones” [P2_Home_1]. For example, the PP dog, although praised for being “something that looks like an animal” [P5_Home_5], was perceived as “dead […] poor old sod” [P5_Home_1]. The activity and movements of Pleo even seemed to reduce some of the dislike associated with its unfamiliar and rubber embodiment for some participants.

He’s the liveliest, fantastic [Pleo]

P7_Home_3

He’s more active [Pleo]

P7_Home_3

Despite the apparent acceptability of JfA cat’s vocalizations and purring, some evidence arose against JfA dog’s vocalizations, through the code *Less Vocalization*. Participants made the following comments about the JfA dog:

Barking aren’t you, you don’t have to bark

P1_Home_3

No barking

[P2_Home_4]

He’s a good animal but he’s not supposed to bark

P2_Home_4

Can’t you shut up?

P2_Home_1

These suggested that the devices makes “a lot of noise” [P4_Home_5], which “would irritate other residents” [P2_Home_4]. While movements and interactivity appeared important, and cat purring was enjoyed, the vocalizations of the JfA dog appeared somewhat undesirable.

##### Embodiment

While familiar animal embodiment was addressed in an earlier theme, here residents provided further insights under the codes *Desirable Esthetics*, *Not too Big or Heavy–Lap Size*, *Soft Feel*, and *Treating as Living Being*. Desirable esthetics were particularly focused on the robots’ eyes and face.

You’ve got a beautiful face you do [JfA cat]

P6_Home_5

Of all devices, Furby had particularly expressive animated eyes.

I like the eyes [Furby]

P6_Home_5

Paro also had large eyes, which appeared appealing.

Those great big eyes, yes those great big eyes [Paro]

P2_Home_2

His eyelashes too! [Paro]

P2_Home_4

Residents also commented on the size and weight of the robots. They generally felt that Paro was “a bit big” [P4_Home_5] and “quite heavy” [P1_Home_4], with a resident saying “[didn’t] like the weight of him […] not for me” [P2_Home_1]. On 1 occasion, Miro and the JfA dog were both described as “too big” [P6_Home_3] and “big” [P3_Home_3], respectively. Further to needing familiar embodiment, appealing face and eyes, and appropriate size and weight, residents very strongly preferred soft furry devices.

I like the fact they’re soft, it’s really nice [Paro]

P3_Home_5

Participants “don’t like […] rubber” [P6_Home_5] or plastic for robot shells, as “you can’t cuddle it” [P11_Home_1]. Interestingly, residents also commented on the feeling of robot insides and stated that they were “really solid [JfA cat]” [P7_Home_3], with the rigidity making the device “look as if he’s dead” [P6_Home_3]. Another resident felt Pleo was “tough as cement inside” [P2_Home_4]. Despite the limitations of available devices, residents very often engaged with the robots as biological beings, and treated the animals as living beings.

Do you like your belly scratched?

P6_Home_5

Residents asked if robots would “bite” [P1_Home_3], and commented they may be “sick” [P1_Home_3], such as when Miro was turned off. A participant even told a device gently “I won’t hurt you darling [JfA dog]” [P1_Home_3], suggesting some attribution of social qualities to the devices.

##### Acceptability

Importantly, the robots seemed to have mainly good acceptability among care home residents, seen through the themes of *Likeability*, *Ownership*, and *Interest in Technology*. Residents demonstrated likability through general positive comments, such as “handsome isn’t he [Paro]” [P3_Home_3] and “he is beautiful [PP Dog]” [P3_Home_3]. Participants generally spent the sessions petting, cuddling, squeezing, and kissing the devices.

I love it, I love the wool [kisses hedgehog 5 times and cuddles tightly]

P8_Home_5

Participants enjoyed the robots very much, and many reported interest in owning or keeping an animal.

He’s mine [PP Dog]

P1_Home_3

Sold, I would like that hedgehog

P11_Home_1

I’d like you in my bed! [Paro]

P8_Home_5

Many residents also spontaneously provided names for the devices, such as “Chatterbox [JfA cat]” [P1_Home_2], “Snowy [Paro]” [P1_Home_2], “Ginge [JfA cat]” [P5_Home_5], and “Lassie [JfA dog]” [P1_Home_3]. The interest of residents in the technology involved in the robots showed some level of understanding of the devices, with participants aware that these are robots or toys, rather than live animals, and yet happy to interact anyway. Participants asked “how does it work?” [P2_Home_2] or “what is the energy source?” [P2_Home_4]. Residents often asked “who made these?” [P1_Home_5] and commented “I’d like to see what’s on the inside of them” [P5_Home_5].

##### Focal Point

The final theme resulting from the analysis was focal point, from the code *Conversations*. This theme represented the time participants spent talking to each other during the free interactions (about the robots), demonstrating that devices can provide a topic to promote conversation between residents. Some examples are included below, but generally, the resulting conversation was humorous and jovial, with 1 focus group erupting into a chorus of “how much is that doggy in the window?”

One conversation was as follows:

P1_Home_5: Mind my cat!

P2_Home_5: It’s a dog darling [laughs]

P1_Home_5: [laughs] I do need to see the optician don’t I!

Another conversation was as follows:

P6_Home_5: He’s laughing at you [Furby]

P8_Home_5: He’s laughing because I’m tickling his belly

P6_Home_5: Oh I thought he was laughing at your face! [laughs]

P8_Home_5: [Laughs] he might be!

### Section 2: Focus Group Results

The results of the focus groups involving residents, family members, and staff are summarized below, although further example evidence is available in Multimedia Appendix 2. Each quote has been assigned a unique identifier, with P representing participating residents, F representing family members, and S representing staff. The graphical representations result from common codes in the data.

#### Preferred Animal

**Figure 3:**
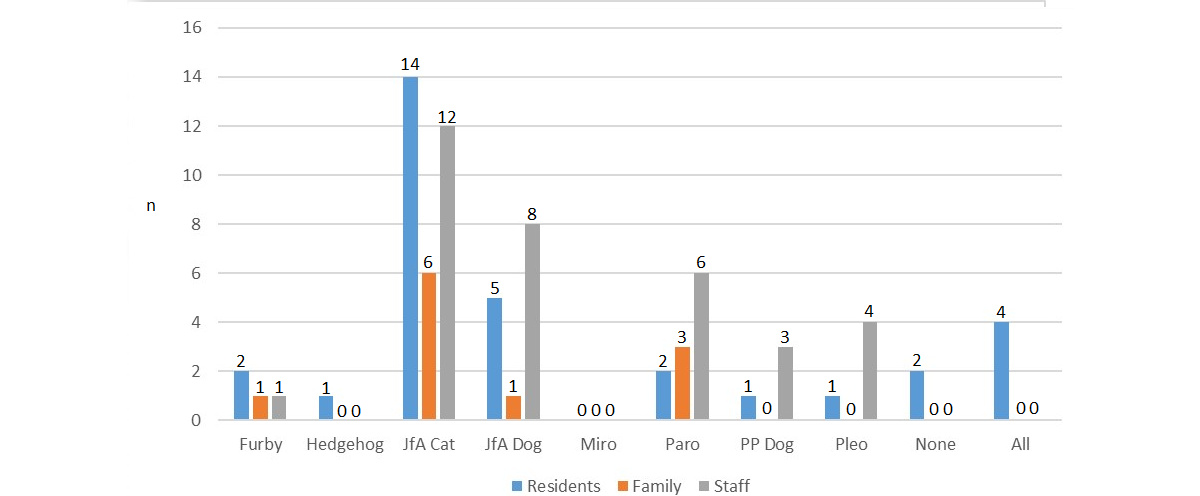
Question 1, preferred device.

Some participants picked more than one device as their preferred device. Residents, family members, and staff all preferred the JfA cat (Figure 3). The JfA dog was the second most preferred device for residents and staff, while Paro was the second most preferred device for family members, also being the third most preferred device for staff.

#### Reason for Preference

**Figure 4:**
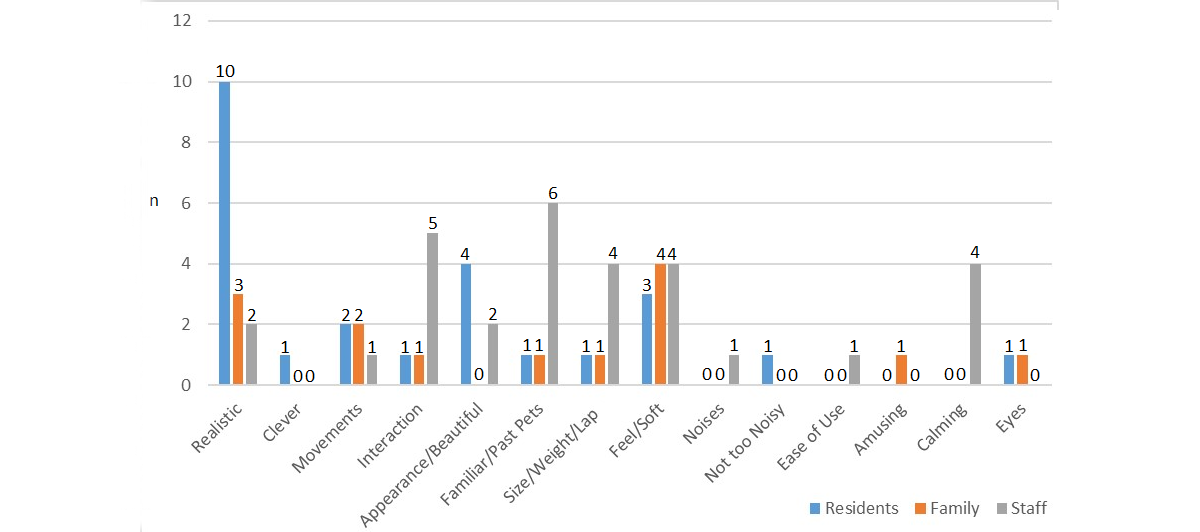
Question 2, reason for preference.

The most common reason for residents selecting their preferred device was it “seem[ed] so real” [P1_Home_2] (Figure 4). Residents may have also been referring to familiar devices as most realistic, suggesting the cat was “very realistic […] not like that seal” [P2_Home_1]. The most common reason for staff preference was that the device represented a familiar animal, such as a cat or dog, as “everybody will stroke a cat or a dog, who strokes a seal?” [S2_Home_1]. For family members, the most common reason for preference was the soft furry feeling, making them “very tactile” [F1_Home_1]. Residents also displayed interest in devices being beautiful and feeling soft, while staff displayed interest in devices being interactive and calming.

#### Design of a New Robot

Participants repeated the importance of a realistic and familiar design, and some design improvements were mentioned alongside measures to enhance practicality. One resident expressed a desire for removing sounds.

No sounds, wakes somebody up.

P1_Home_3

This was supported by a family member who felt robots could sense when to be quiet.

When they have their snooze and they drop off, it drops off and doesn’t disturb them.

F3_Home_1

Desirable features also included being “something warm, purring on her lap” [F1_Home_5]. A staff member felt “breathing is good” [S1_Home_2]. Participants also valued the device turning “its head towards you” [F1_Home_5] and appearing to provide attention. Some family members and residents desired command responses, such as “the dog should sit up and beg” [P3_Home_3] or “wanting to play like a dog” [F1_Home_2].

For the physical body, participants discussed “the weight” [S8_Home_4], as “it could be a bit lighter” [F1_Home_4]. This links with being “the right shape to go on their lap, the cat is perfect to go on a lap” [S1_Home_3]. Devices that are “too heavy,” such as “Paro,” may be less “accessible” for “older people [who] are quite frail” [S1_Home_3]. Participants felt future devices should certainly “look like something [residents] had in the past or it will be alien to them” [S10_Home_5]. One staff member also felt they could be “softer […] in the body” rather than feeling “robotic” under the soft surface [S2_Home_2].

For practicality, it was noted the devices should be “robust” [S1_Home_1]. A number of participants also requested robot “covers come off” [S2_Home_1], as “it needs to be washable” [S6_Home_4 and S5_Home_4]. Family members also commented “the fabric […] can you take it off and wash it? Because […] they’re old and it gets greasy and mucky” [F3_Home_1], which “could see it getting quite dirty after a while” [F2_Home_1].

#### Abilities for a New Robot

Residents agreed that the abilities of a new robot should include being “interactive” as “that’s the idea of a robot” [P2_Home_1] and valued when it “talks at me and he looks at me” [P5_Home_1]. The importance of interactivity was supported in criticism of the PP Dog, as “you want it to play, a bit more action” [P11_Home_1]. Staff and family members agreed it should “respond to her” [F1_Home_5] and “it’s got to be interactive […] so residents have something to have their minds think about” [S2_Home_2].

Command responses were mentioned again.

It would be nice if it could say […] roll over or beg

S1_Home_1

If you tell it to stop moving or sit or something it gives them vocabulary they might have forgotten.

F2_Home_1

The use of warmth was mentioned again with the comment “kind of like temperature, like warmth” [F1_Home_2]. Eye contact or perceived attention was certainly praised with the comment “looking for them […] the heads moving, eyes opening and closing” [S1_Home_3]. Such movements involve fairly simple technology, and staff felt “[Paro is] probably too complex really for [residents] needs” [S1_Home_1].

The possibility for command responses for some residents could be solved through the suggestion of making a device “adaptable to the person” where the pet could be “peaceful and relaxing […] but do other things when needed” as “if you’re gonna make something make it wide ranging, make it as adaptable as possible” [S10_Home_5].

#### Feel

**Figure 5:**
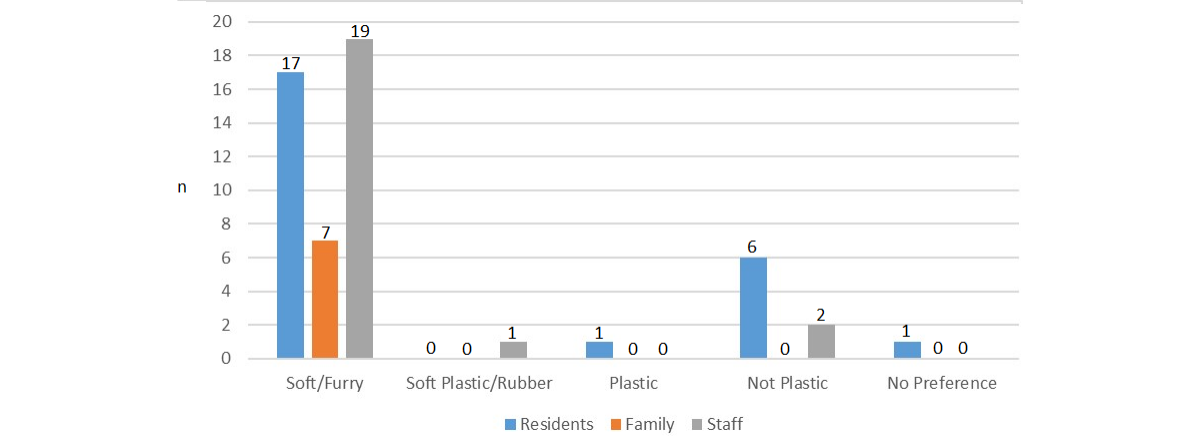
Question 4, feel of the device.

All categories of participants supported soft furry embodiment for future robot designs (Figure 5), which were considered “pleasant to touch” [S2_Home_1], and “they could stroke it” [S2_Home_1], which was “more therapeutic” [S1_Home_5]. Plastic or rubber was not generally desired as “you don’t get rubber animals” [P6_Home_5] and it could “be too cold” [P2_Home_5]. One resident liked all the robots and felt “the rubber one interacted anyway so I’ve got no preference” [P7_Home_5].

#### Expressions and Behaviors

Participants talked of the importance of “facial” [F1_Home_2] expressions being the “first thing” [F2_Home_2] that “people look at” [P2_Home_4]. Staff also felt it “would be quite good” if “eyebrows move and eyelids move” [S1_Home_2]. Linked with facial expressions was the appearance that the device is looking at the user.

It’s got an expression and it looks at you.

P2_Home_1

The looking, that sort of interaction.

S5_Home_4

To look towards you.

S12_Home_4

This is also related to the importance of eye design.

The eyes, the eyes.

P1_Home_4

See the eyes moving.

P2_Home_4

Moreover, breathing and “purring” [P6_Home_5] were praised.

Once she realized it was breathing, she was like aw, she wanted to listen.

S1_Home_3

The breathing is relaxing.

S5_Home_5

I love to hear them purr.

P8_Home_5

Purring was considered useful for those with hearing impairments, as “you can feel the cats purring even if they can’t hear it” [S8_Home_5]. Further behaviors enjoyed included the cat “rolling over” [S10_Home_5], as “their movement is what makes them look real” [P7_Home_5] and “more interesting” [P2_Home_5]. Command responses were mentioned again, such as “give me a paw” [F1_Home_1].

The animal demonstrating its mood was considered important, possibly through known behaviors, such as “wagging the tail for the dog […] cat purring” [F1_Home_1], or possibly through lights, where a device may “light up to show their mood” [S2_Home_2]. Generally, participants felt the device should appear “happy” [F1_Home_2], but could indeed be adaptable depending on the resident’s needs, so robots could be set on a “chilled, or happy, placid” [S2_Home_2] mood, depending on the need of each resident.

#### Design Features to Avoid

**Figure 6:**
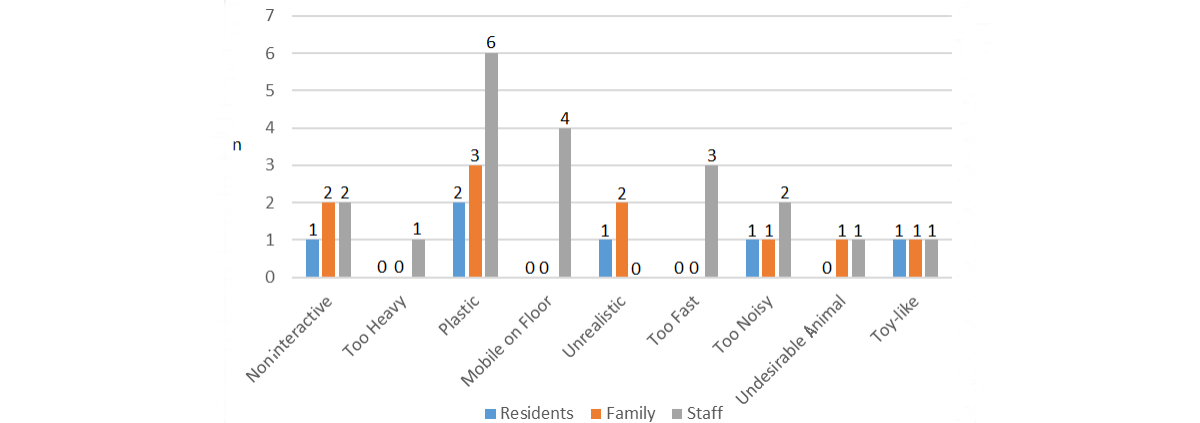
Question 6, features to avoid for a new robot.

Design features to avoid received fewer responses (Figure 6), likely due to discussion elsewhere, but the feature most commonly mentioned by residents, family members, and staff to avoid was plastic embodiment. Staff also felt it was important robots were not autonomous and mobile on the floor, which could cause “hazards” [S6_Home_4]. It was also felt that the devices should not move “too quick” [S5_Home_4], or be vocalizing “too loud” [F1_Home_4] or “all the time,” as it could “irritate the other residents” [P2_Home_4]. Participants felt the design should avoid being toy-like, with Miro, Pleo, and Furby described as “childlike” [P5_Home_5]. Family members felt residents may “take offence” [F1_Home_4] at being given robots that resemble toys too much, comparing toy-like robots to “children’s puzzles” [F1_Home_4].

#### Talking

**Figure 7:**
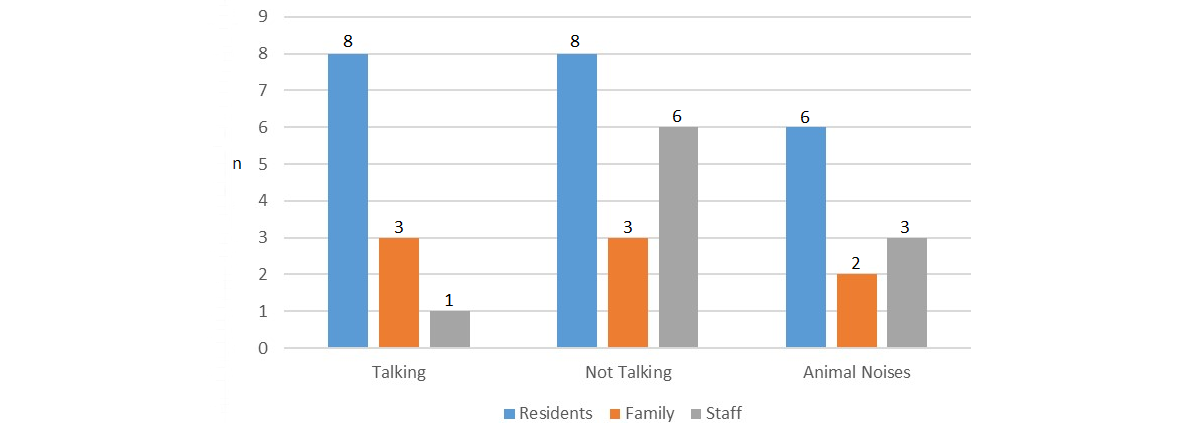
Question 7, opinions on a new device speaking human language.

There was no definitive answer on a device speaking human language (Figure 7), although combining *not talking* responses with *animal noise* responses would suggest talking was far less desirable, but still of interest to a number of participants. Reasons for desiring speech included the potential for “speech therapy” [F1_Home_1] to “encourage [residents] to speak” [F2_Home_1]. Some staff felt residents “might be able to express their feelings more than what they can do to a carer or doctor” if the robot spoke [S2_Home_2]. Some residents felt it would be “wonderful” [P6_Home_1] and would “like it if he spoke back” [P1_Home_3], as it “would be very interesting” [P2_Home_5]. However, other residents responded “I’d say you were nuts and I was nuts, round the bend good and proper” [P5_Home_1]. Family members and staff also worried it was “just too weird” [F1_Home_2], or could cause “sensory overload, like processing why is a cat talking to me” [S1_Home_3] and even be “disturbing” [S6_Home_4].

#### Personalization

Most participants were generally positive about personalizing devices, and being able to choose “your own color” [P2_Home_1], “which animal I’d like” [P6_Home_1], or even “a pet they’ve had in the past” [S1_Home_2], which may “spark something off” in their memory [F4_Home_4]. Some participants felt the available devices needed no improvement however, as “they’re done well enough aren’t they” [P11_Home_1]. Staff worried about personalizing robots not being “cost effective” [S1_Home_2]. They commented “that robot is going to be personable to them […] everyone’s going to have different opinions” [S1_Home_1] and “when that person’s gone, that animal is not going to be significant for anyone else,” but then stumbled across the idea to “change the outer” [S1_Home_2], therefore allowing customizability with “a robotic framework that goes into every animal, and then a shell you could change” [S1_Home_3]. Having individual covers would also mean covers would be “washable” [S1_Home_5]. Having residents involved in creating the shell was also interesting, with staff suggesting residents could “knit and crochet” [S2_Home_2] to create something like the handmade hedgehog. Being involved and either creating or personalizing the device “would feel like they’re part of something” [S2_Home_2] and would help them “get more attached” to the device [S3_Home_2].

#### Realistic and Familiar

Participants discussed both the concept of it looking “realistic” [P8_Home_5] and “life-like” [P4_Home_5], further to being a familiar animal “they can relate to” [S1_Home_3], particularly a “domestic animal […] I don’t know whether the seal would go down as well” [F2_Home_2]. All groups generally supported more realistic and familiar embodiments, with Miro described as “too futuristic” [S1_Home_2], and Paro felt incongruent in the setting.

Why have you got a seal in a home, you wouldn’t.

F1_Home_1

In contrast, familiar animals received the following comments:

It’s easy to identify with the cat.

F1_Home_1

It’s more therapeutic if they recognize it.

F3_Home_4

Everybody […] will stroke a cat or a dog.

S2_Home_1

The benefits of a familiar animal included that it “stimulated their memories” [S2_Home_1], as it represented “something I recognize” [P2_Home_5]. Unfamiliar and unrealistic forms were considered “better for people with learning disabilities” [S6_Home_5] or “children” [F4_Home_4]. A very small number of residents displayed interest in unrealistic embodiment as “it would hold your gaze because it’s different” [P2_Home_5].

#### Keeping a Robot

For Question 10, participants were asked if we could leave a device behind for the benefit of residents, which one (if any) would they want left.

**Figure 8:**
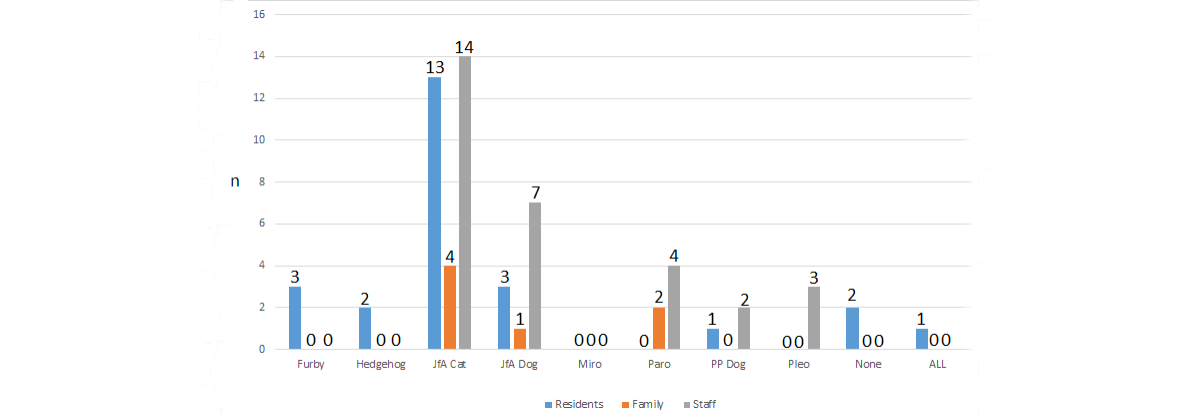
Question 10, which device would participants keep for residents use.

Similar to Question 1 where preference was shown for the JfA cat and dog, the combined choices of participants favored keeping the JfA cat, followed by the JfA dog (Figure 8). In total, 47 participants responded to this question, and 45 of them agreed to keep a device, with only 2 participants responding “no” [P7_Home_5]. Some family members and staff chose to keep Paro, but this device was not selected by residents.

#### Summary

The most preferred and most likely to be adopted devices were the JfA cat and JfA dog. Based on the focus groups and free interactions, the combined evidence has produced a *recipe* for future robot pets aimed at care home residents, based on the user-centered inputs of residents themselves, as well as their family members and care team. The requirements are as follows: (1) appear familiar and realistic (dog or cat) to avoid infantilization, (2) be soft and furry, (3) look at the user, blink, show expressions, and have engaging eyes, (4) breath, purr, and be warm (tactile responses for those with hearing impairments), (5) be of suitable size and weight for laps, (6) have adjustable volume and frequency of noise and vocalizations, (7) have removable skin for cleaning, (8) have a customizable appearance, (9) possibly respond to commands, (10) possibly have more realistic robot *insides*, (11) possibly sense when to shut down, (12) possibly adapt to the need of each user (eg, displaying certain moods dependent on the requirement to calm, sooth, or entertain), and (13) have the ability to talk (further research).

## Discussion

### Overview

This work has provided important insights into the views of care home residents, family members, and care staff regarding the design and use of companion robot pets. This work demonstrates an overall good acceptability of robot pets, with the majority of residents, family members, and staff selecting a preferred device and suggesting they would keep a robot if they had the opportunity to do so. This work also highlights some interesting design considerations.

### Principal Results

Evidence suggests the most important design requirements to be familiar animal embodiment and a soft furry shell, congruent with previous work [27,32]. However, some participants, including residents, reported that feel was less important than interactivity, with the lively interaction of Pleo creating positive appeal despite an undesirable rubber shell. Interestingly, further to a soft shell, participants expressed an interest in warmth. Desire for such tactile features may relate to the human use of touch as a primary nonverbal communication channel [40]. Social touch has an important role in prosocial and bonding behaviors, even between humans and robots [40]. Human skin has specific receptors to process affective touch [41], and therefore, tactile feedback provided by robots is a key consideration for future developments. An additional feature discussed in this work, which was unexplored previously, is the feeling of robot *insides*. Participants felt the JfA cat was somewhat rigid, and other residents commented on the hard-feeling robotics under the soft exterior of devices. Thus, there may be value in improving the *feel* of the insides of robots, further to the shell, by perhaps providing extra padding for softness or replicating a realistic body frame.

Regarding familiarity, we know Paro was designed with unfamiliar seal embodiment to avoid expectations [42]. Paro is the most well researched companion robot [10]; however, Moyle et al [8] previously saw considerable variation in older adults’ responses to Paro during a randomized controlled trial, with some residents rejecting the seal. Our work suggests that this may result from the unfamiliar embodiment of Paro, as residents in our study sometimes rejected Paro, alongside other unfamiliar devices (Pleo and Miro), whereas the best acceptability and preference were shown for familiar devices that represented domestic pets (JfA cat and dog). Some devices were perceived as more suitable to children, perhaps because they were more toy-like (bright colored Furby or rubber Pleo), or because they were unfamiliar embodiments that would not usually be found in a care home, thus being obviously a toy. This should be investigated further to consider any impact of unfamiliarity or toy-likeness on perceived infantilizing. Any evidence of infantilization would support the ethical concerns raised by Sparrow and Sparrow [43] on the inappropriateness of robot pets for older adults. Several residents in this study reported feeling “nuts” or “like fools” for interacting with such devices, although this is not necessarily a negative response and would need further exploration. Family members showed some disagreement with unfamiliar devices, and felt residents may take offence, with 1 comparing toy-like robots for older people to children’s puzzles. Use of familiar and recognizable embodiment thus seems important for older adults to enhance positive response and recognizability, and reduce risks related to possible infantilization and rejection. Further to being familiar, participants also desired robots to appear as realistic and life-like as possible, as realistic embodiment appeared to reduce perceptions of devices as toys.

The use of familiar and realistic animal embodiment may conjure ethical concerns on deception, if a robot appears too similar to a living creature. Previously, Sparrow and Sparrow [43] suggested enjoying robot pets required people to deceive themselves as to the realness of the interaction; however, care home residents in this study showed good acceptability of robots despite awareness and interest in the devices’ technology, thus being aware of their nonliving nature. Conversely, residents did treat robots as living beings, and our sample consisted only of residents with the capacity to consent. It is possible residents with dementia (without capacity) may indeed be deceived as to the real nature of such devices [44]. A few family members raised ethical concerns toward their relatives interacting with robots, particularly unfamiliar ones (eg, comparing robots to children’s puzzles), and a resident’s relative did present some opposition in previous work [23]. While residents did not directly report offense, they did suggest that unfamiliar devices were more “childlike.” The ethical considerations of companion robot use thus requires further enquiry, particularly considering familiar and realistic devices, which may be even more deceptive than devices such as Paro. We have discussed the ethical considerations of robot pets elsewhere [45].

With regard to robot appearance, eye and face design seemed particularly important, as did the device appearing to look at the user. As mentioned by participants, residents naturally look at the face and eyes the most, and participants appeared to prefer “animated” and “big” eyes. Regarding robot body size, this research confirmed that Paro is indeed too big for older people, as noted previously [10]. Participants reported that residents are often slight and frail, and commonly engage with robots on their laps, with Paro being too heavy for comfortable use. Likewise, the upright position of the JfA dog was not considered the best suited to the lap. The negative response to Paro’s size and weight seen here may help further explain some negative reactions to Paro in previous work [23], combined with evidence against the use of unfamiliar devices, which can shed doubt on the continued selection bias for Paro in companion robot research [9,10].

Regarding interactivity and displayed behavior, Moyle et al [8] suggested previously that Paro was more engaging than a plush toy. Here, stakeholders confirmed the requirement for movement and interactivity from a companion robot, viewing inanimate options as ornaments and pretty things rather than companions, thus implying that movement/interactivity produced the perception of a social entity. However, the level of interactivity required remains uncertain. Participants in this study reported preference for the JfA cat and dog, as in our prior work with a smaller more independent sample of older adults [24], suggesting that devices less sophisticated than Paro may prove to be adequate companions. The JfA devices respond only to touch and sound, with a limited range of set movements, in comparison to Paro’s artificial intelligence, range of sensors (including touch, sound, light, and position), and bespoke responses. Indeed, 1 member of the staff reported that Paro’s technology was too complex for this client base. Generally, desired movements included looking toward the user, rolling over, wagging the tail, being expressive, breathing, and possibly feeling warm. There was some interest in command responses, and there were indefinite opinions on robots talking, as seen in our prior work [24]. However, a limitation of exploring the interactivity requirement in this study is the short interaction time. It is possible that more sophisticated technology and interactions would hold engagement better over longer-term use. Some research exists on the longitudinal use of Paro [13], but literature is generally limited for more affordable devices, reducing our understanding of the interactivity required for long-term engagement. Although one of our other studies indicated no novelty effect of affordable pets over 6 months, the research included only 2 implementation sites [46], leaving scope for further exploration of longitudinal studies on affordable robots. A further limitation of exploring interactivity in this study is that our sample included only residents with the capacity to consent, who are less likely to have a diagnosis of moderate or severe dementia. A sample of residents with moderate to severe dementia may respond differently to interactive robots.

Another aspect related to robot behavior is vocalization. Previously, Robinson et al [23] suggested that Paro’s vocalizations may be distressing for residents of a dementia unit. In this work, we also found that residents did not like loud or frequent vocalizations, particularly the barks and yaps of the JfA dog. In contrast, during focus groups, many residents commented on the value of the purring and vocalizations of the cat, and participants in all stakeholder groups commented positively on devices making animal noises. Some residents even discussed the value of auditory responses for older adults with sight impairments. This factor clearly requires further specific enquiry on the acceptable type, frequency, and volume of vocalization.

This study has thus contributed toward user-centered discussions on embodiment, behavior, interactivity, and vocalizations, although further enquiry is needed. Some additional interesting features also arose. Particularly, the interest in breathing and warmth meant life-simulation features should be considered for inclusion in future work. Further interesting discussions arose on removable fur for hygiene purposes due to concerns on shared objects becoming unclean. Although this study was conducted prior to the COVID-19 pandemic, infection control considerations for shared robots in care homes are particularly relevant in the current context [47,48].

The study also hinted at some potential benefits of robots, despite the short interaction time, on communication, in particular through the theme of *robots as a focal point*. This is congruent with the conclusions of a recent scoping review on the impacts of affordable robot pets [49]. In our study, it is quite possible that residents engaged in additional conversations around robots as they were new and exciting, again demonstrating the requirement to assess any novelty affect [50], furthering our exploratory prior work with affordable pets [46], and the limited number of available impact studies with such devices [49,51].

### Strengths and Limitations

The strengths of this work include the participation of 3 stakeholder categories (residents, family members, and care home staff) and the consideration of responses based on first-hand observations, thus ensuring informed opinions. While views between stakeholder categories can vary [28], our work found that residents, staff, and family members had good congruence in responses for many features. The most variation was seen for robots talking, with residents and family members less decisive, while staff responded more negatively to robots speaking. Perhaps this represents an area of unmet need underestimated by care staff. Future work could explore this further, but it is likely that the most weight should be applied to the end-user perspective. While acceptability among wider stakeholders is essential for devices to be procured, facilitated, and maintained, the perception of the end-user on functions is likely of most importance. Future work may also seek to expand the stakeholder categories even further to include independently living older adults and compare perceptions on robot preferences for more able older adults. This work also responded to an identified literature gap regarding the lack of companion robot comparison studies [9]. Previously available comparison studies focused mainly on the input of care providers, and lacked Paro as a comparator [27] or the use of companion robots designed for older adults [32]. These limitations were responded to in this work. A further strength of this work in comparison to previous studies is the randomization of robot presentation order. The serial position effect theory suggests that the first and last presented items may be better remembered than those within the sequence (primacy and recency effect) [52], and particularly when working with older adults who may experience some cognitive impairments, the method of presentation requires additional consideration. For this reason, we randomized the order of robot presentation and represented all robots together during the focus groups to ensure that all devices were recorded in the short-term memory for discussion and comparison. As already discussed, the limitations of this work include the short interaction time and the inclusion of only residents with the capacity to consent. The short interaction time may have resulted in a novelty effect, meaning longer-term studies with more affordable devices are required to explore longitudinal engagement. Future research may also consider other devices that fit the design requirements stated here, but with additional functions to explore, such as the JustoCat. Another possible limitation is that we focused on explicit design preferences, rather than long-term engagement or well-being outcomes. However, in line with the user-centered approach [25], an understanding of user requirements is the essential first step in user-centered design. Based on the results of this study, future robot developments may more accurately match user requirements and provide more consistent results. Additionally, the acceptability and preference of affordable JfA devices provide scope for future research considering these pets in long-term trials for assessing their impact on well-being.

### Conclusion

Care home residents, family members, and staff were all generally open and accepting of the use of companion robot pets, although a very strong preference was shown toward the JfA cat and dog, due to the familiar embodiment. Participants discussed many design features, with soft fur, interactivity, nice eyes, and movements appearing important. Unfamiliar embodiment and appearing toy-like produced fewer positive responses. Further work is required for feature prioritization, and to achieve a greater understanding of suitable sizes and weights for such devices. As this work suggests strong acceptability of affordable JfA devices by residents in care homes, further work is required to explore the use and impact of devices, such as these familiar robot pets, in this setting.

## Appendix

Multimedia Appendix 1 Further evidence from free interactions.

Multimedia Appendix 2 Further evidence from focus groups.

## Abbreviations

ADLs: activities of daily living
JfA: Joy for All
PP: Perfect Petzzz
