# Supplementary material for: User-Centered Design of Companion Robot Pets Involving Care Home Resident-Robot Interactions and Focus Groups With Residents, Staff, and Family: Qualitative Study
Source: JMIR Rehabil Assist Technol. 2021 Nov 1;8(4):e30337. doi: 10.2196/30337 (PMC8593804; doi:10.2196/30337)
Supplement: Multimedia Appendix 1 [file rehab_v8i4e30337_app1.docx]

**Multimedia Appendix 1.** Further evidence from free interactions.

| **Theme** | **Codes** | **Example Evidence** |
| --- | --- | --- |
| **Familiar** | **Plastic and Unfamiliar as Infantilising** | “[Laughs at JfA dog barking] this is crazy!” (P1_Home_4)  “Have you shown these ones to children? [Miro]” (P1_Home_5)  “I’m talking to him, we must be crazy! [Miro]” (P7_Home_5)  “You’re making fools out of us, do you know that? [Paro]” (P4_Home_5)  “[Looks at seal] [laughs] you’re joking” (P5_Home_5)  “They’d be lovely for children, because children have got vivid imaginations” (P2_Home_5)  “More appropriate for young children, they’d love this [Paro]” (P2_Home_5)  “The seal is lovely and ideal for a young child” (P2_Home_5)  “[Laughs] that’s very amusing isn’t it [Miro]” (P3_Home_5)  “I should think that’s something from outer space [Miro]” (P2_Home_5)  “Is he off the moon [Miro]” (P2_Home_5)  “Ohhh I know, I know you’re beautiful.. you’re making me look stupid do you know that! [JfA dog]” (P4_Home_5)  “Steady monster [Miro]” (P2_Home_5)  “Very soft, quite pretty too [Furby], good for somebodies children” (P2_Home_4)  “This one would be popular with young children [Miro]” (P2_Home_4)  “Younger child would like to play with these [Miro]” (P2_Home_4)  “[Laughs] you’re like kids all of you!” (P1_Home_4)  “People will think I’m stupid if they see me now [JfA dog and Paro]” (P1_Home_2)  “They’re amusing, they’re not a silly thing” (P3_Home_5)  “Well, that’s alright for children [Pleo]” (P5_Home_1)  “My great granddaughter would love that [Pleo]” (P11_Home_1)  “A tiny little boy might like [Miro]” (P11_Home_1)  “I should give a child something like this [Furby]” (P6_Home_1)  “That’s lovely for children, I should imagine [JfA dog]” (P5_Home_1)  “You’re beautiful aren’t you, not saying very much […] we’re all mad, what are all these people here, eh? (Paro)” (P5_Home_3)  “[Stroking Paro] We’re nuts! We’re nuts!” (P5_Home_3) |
|  | **Prefer Familiar** | “I’m not keen on any except the little one [Perfect petz]” (P4_Home_5)  “Well, nobody could love you like your mother could they [to Pleo], no no no, I’m sorry” (P1_Home_5)  “It’s not the sort of creature you’d find in a home [Paro] but it’s still my favourite because it’s so soft” (P3_Home_5)  “This one could be real [Perfect petz], something that looks like an animal” (P5_Home_5)  “The cats very good” (P2_Home_5)  “I prefer the more natural things, the best one is that cat” (P1_Home_4)  “She’s looking so worried! [about Pleo]” (P2_Home_4)  “Oh well you live in the water and I hate the sea [Paro]” (P4_Home_5)  “I don’t know that I particularly like that. I don’t like that […] I don’t like it because it’s blue [Furby], but I do like the others” (P5_Home_3)  “[Dislike] because it’s not natural [Paro]” (P7_Home_3)  “Most unusual isn’t it [Hedhehog]” (P5_Home_3)  “The dog is lovely, I like dogs, we had all sorts of dogs” (P3_Home_3)  “[holding dogs] we used to have dogs, I have a picture of my mum and dad with our dog” (P1_Home_4)  “It’s lovely [dog] but I like the cat, I’ve had a dog all my life, I’ve only ever had one cat” (P4_Home_5)  “I’ll have you for tea tomorrow night [Paro]” (P1_Home_1)  “I suppose really you’re quite a beautiful animal, to think they skin you to make a coat [Paro]” (P4_Home_5)  “What is that, a baby seal? […] You know, baby seal, they are skinned alive when they are born” (P5_Home_1) |
|  | **Unfamiliar are Unrecognisable** | “What’s it supposed to be? Bat? [Furby]” (P1_Home_5)  “Oh look, is that, I think it’s a seal? Making eyes at me [Paro]” (P1_Home_3)  “The only think, you look at that, see that [hedgehog] and you wonder what on earth, you know, bit strange” (P4_Home_3)  “[hedgehog] it could be a duck” (P1_Home_3)  “[shown Pleo] oh that’s a lamb, that’s a baaa lamb” (P1_Home_3)  “I've never seen anything like this before [Pleo and Miro].” (P6_Home_1)  “Is that a seal? [Paro]” (P6_Home_3) |
|  | **Robot Rejection** | “I don’t want it [Furby]” (P4_Home_3)  “[offered hedgehog] no” (P4_Home_3)  “[Shown pleo] [bats it away] not for me” (P4_Home_3)  “[Offered dinosaur] not particularly” (P2_Home_1)  “Don’t sit him by me, he might eat me [Pleo]” (P5_Home_1)  “What is it? [Furby] […] very clever but I wouldn’t want it” (P11_Home_1)  “Don’t like the other ones. No good at all. They’re not good at all [Has dog]” (P5_Home_1)  “I wouldn’t want him [dinosaur], I like the other one [cat]” (P6_Home_1)  “the white one I wouldn’t go for. I don’t know. She’s a bit, no, there’s nothing to encourage me to touch it. No I couldn’t do it. No I would go away from it [Paro]” (P5_Home_3)  “[shown Pleo] I wouldn’t like to” (P5_Home_3)  “It’s like a toy, I don’t know that I’d want it as a toy [Miro]” (P5_Home_3) |
| **Robot Actions** | **Important Expressions and Behaviours** | “I like him actually, I think because of his activity and his response it’s like he’s talking to you” (P5_Home_5)  “They’re almost alive aren’t they” (P1_Home_5)  “I think they’re ideal for blind people, ideal” (P2_Home_5)  “[Cat meows] ideal for somebody who is blind” (P2_Home_5)  “The cats very good isn’t it, active, this isn’t so active [JfA dog]” (P3_Home_5)  “Look at him, he’s moving his face [JfA dog]” (P3_Home_3)  “[cat rolls] yeah, oh yeah, oh! Meow, meow, meow, be good, be careful, you’re alright” (P1_Home_3)  “[cat rolls] oh my goodness!” (P4_Home_3)  “You’re talking to me aren’t you [JfA dog]” (P1_Home_3)  “He’s talking to me [Furby] don’t swear” (P1_Home_3)  “Oh a lot of fun you are, funny boy [Furby]” (P2_Home_5)  “Nice animated eyes, that’s really special [Furby]” (P3_Home_5)  “Hetty (hedgehog) is just an ornament really. I like the movement ones. And the cat in particular was absolutely gorgeous” (P2_Home_1)  “Hear her purring!” (P5_Home_1)  “When you talk, it will answer. When you talk it will answer, because it can hear the vibrations from your voice. That's why she answers.” (P5_Home_1)  “Oh look at the eyes closing” [Paro]” (P1_Home_4)  “The eye blinking is lovely [Cat]” (P2_Home_4)  “One of them, he’s sleeping all the time [perfect pets] […] does it work?” (P5_Home_3)  “He’s the liveliest, fantastic [Pleo]” (P7_Home_3)  “He’s more active than the other one [Pleo]” (P7_Home_3)  “I should think he’s dead, the way he’s lying. Poor old sod. Never mind my love [Perfect pets]” (P5_Home_1)  “I don’t think they do wake up [Perfect petz]” (P2_Home_1)  “Is he alive or sleep [Perfect petz]” (P5_Home_1)  “He doesn’t wake up more than that does he? [Perfect petzzz]” (P6_Home_3)  “It’s a bit annoying, you want to know what he’s saying [Furby]” (P2_Home_1) |
|  | **Less Vocalisations** | “You’ve got an awful lot to say for yourself young man [Miro]” (P2_Home_5)  “You make a lot of noise [JfA dog]” (P4_Home_5)  “I think the barking would irritate the other residents” (P2_Home_4)  “Barking aren’t you, you don’t have to bark” (P1_Home_3)  “No barking” (P2_Home_4)  “He’s a good animal but he’s not supposed to bark” (P2_Home_4)  “Can’t you shut up?” (P2_Home_1)  “I quite like him actually [picks up Miro] Oh! Heavy! Quite noisy” (P2_Home_1) |
| **Embodiment** | **Desirable Aesthetics** | “What are you called? You got a beautiful face you do [JfA cat]” (P6_Home_5)  “I like the eyes [Furby]” (P6_Home_5)  “Lovely work! Very clever! [Hedgehog]” (P1_Home_4)  “It’s a nice thing, to hold [Hedgehog]” (P2_Home_4)  “His eyelashes too! [Paro]” (P2_Home_4)  “Those great big eyes, yes those great big eyes [Paro]” (P2_Home_2)  “I think the cat looks real. That looks real” (P11_Home_1)  “I love this hedgehog” (P11_Home_1)  “Cats beautiful” (P2_Home_1)  “Whose this one? [Furby] I like his coat, the colour, I like it” (P1_Home_3)  “Beautiful eyes, great big eyes [cat]” (P5_Home_3)  “Something about their eyes. Beautiful [JfA cat]” (P6_Home_3) |
|  | **Not too big or heavy - lap size** | “It is a bit big [Paro], but that one certainly appeals to me” (P4_Home_5)  “This one’s too big, takes up too much room [Paro]” (P4_Home_5)  “Big isn’t he? [JfA dog]” (P3_Home_3)  “Quite heavy [Paro] I’ve never seen anything like it, I think they’re very good, they’re so realistic” (P1_Home_4)  “He’s quite heavy isn’t he [Paro]. No not for me [hands seal back]. I don’t like the weight of him” (P2_Home_1)  “Very heavy, a bit of an armful [Paro]” (P2_Home_1)  “I quite like him actually [picks up Miro] Oh! Heavy! Quite noisy” (P2_Home_1)  “His body is heavy […] he doesn’t feel real [Paro]” (P5_Home_1)  “Quite heavy [Seal]” (P7_Home_1)  “He’s too big [Miro]” (P6_Home_3) “Yeah he is really isn’t he” (P7_Home_3) |
|  | **Soft feel** | “Oh how odd, it’s rubber! I don’t like it rubber” (P6_Home_5)  “I like the fact they’re soft, it’s really nice [Paro]” (P3_Home_5)  “Feels like rubber skin of a kind, and tough as cement inside I should think” (P2_Home_4)  “Oh the feel! [Paro]” (P1_Home_3)  “You can’t cuddle it [Miro]” (P11_Home_1)  “No, I like the fur [Miro]” (P1_Home_4)  “No I don’t like that feeling [Pleo]” (P1_Home_4)  “You’re all rough aren’t you? [Pleo]” (P1_Home_2)  “I aren’t so much for him, he looks as if he’s dead [JfA cat]” (P6_Home_3) “He’s really solid [JfA cat]” (P7_Home_3) |
|  | **Treating as Living Being** | “it won’t bite will it?” (P1_Home_3)  “Do you like your belly scratched eh? You’re a cheeky thing” (P6_Home_5)  “Oh it’s lovely, he likes that [strokes cat under chin]” (P2_Home_5)  “he’s obviously gone to sleep [breathing dog]” (P1_Home_3)  “[Strokes dinosaur] don’t bite, no don’t bite, good boy, good boy, good, yeah nice, he’s winking his eye” (P1_Home_3).  “He don’t, he doesn’t bite? [Miro]” (P1_Home_3).  “That is like having a breakfast [Pleo]” (P1_Home_3).  “Maybe sick [when Miro not active]” (P1_Home_3).  “Oh pussy cat, it’s a she isn’t it?” (P1_Home_3)  “Go to sleep if you want [JfA dog]” (P1_Home_3)  “Go meow and go to sleep [JfA cat]” (P1_Home_3)  “Does it like having a wash?” (P1_Home_3)  “[to JfA dog] don’t chase the cat! If you do they’ll scratch you, then you’ll bleed, we can’t have that can we? No, good boy” (P1_Home_3)  “He’s been eating too much I expect [JfA cat]” (P1_Home_3)  “I won’t hurt you my darling [JfA dog]” (P1_Home_3)  “They like their necks tickled don’t they [Pleo]” (P6_Home_3)  “You watch he don’t do something when he lifts his tail” (P5_Home_1)  “He’s blinking his eyes, are you comfortable” (P2_Home_4)  “Oh yes that’s very much like a seals cry” (P2_Home_4)  “He likes it underneath the chin [Paro]” (P2_Home_4)  “You want your belly stroked don’t you” (P1_Home_4)  “They respond the way they should, I’m stroking him away [JfA cat]” (P1_Home_4)  “Hello, you’re back again, want your tummy rubbed [JfA cat]” (P2_Home_5)  “He can hear what you’re saying, did you hear that, that’s not fair is it what [resident] said, poor sausage” (P5_Home_5) |
| **Acceptability** | **Likability** | “they’re lovely aren’t they” “he is beautiful [breathing dog]” (P3_Home_3)  “Lovely it is, you’re beautiful aren’t you? Yes you are [breathing dog]” (P1_Home_3)  “Handsome isn’t it, oh look at that” All that hair […] isn’t that lovely?” (P3_Home_3)  “Oh look at him. Isn’t that lovely [Miro]” (P3_Home_3)  “Look at that, it’s beautiful, it’s such a big beautiful. I think that’s lovely” (P3_Home_3)  “Handsome isn’t he [Paro]” (P3_Home_3)  “[Cat purring] isn’t he beautiful, I could keep it like that, if it’s trouble you can take them home again” (P3_Home_3)  “They’re beautiful. Yes, they are.” (P6_Home_1)  “A lot of work gone into these” (P11_Home_1)  “You are beautiful [cat], and you [dog]” (P1_Home_2)  “Isn’t that wonderful [Paro]” (P1_Home_4)  “Very nice, very well made” (P1_Home_5)  “Lovely, isn’t it? [JfA Cat]” (P2_Home_5)  “You are quite beautiful” (P4_Home_5)  “Very cute” (P5_Home_5)  “Lovely girl aren’t you, you’re obviously a lady [JfA cat]” (P5_Home_5)  “Lovely” (P6_Home_5)  “They’re uncanny really aren’t they” (P7_Home_5)  “They’re lovely” (P7_Home_5)  “Oh they’re lovey, gorgeous you are, oh I could sit here all day” (P7_Home_5)  “you purr, you purr, lovely” (P7_Home_5)  “I love it, I love the wool [kisses hedgehog five times and cuddles]” (P8_Home_5) |
|  | **Ownership** | “He’s mine [breathing dog]” (P1_Home_3)  “Blue eyes, [Furby], lets call him Frank Sinatra” (P6_Home_5)  “You’re my puppy aren’t you [JfA dog]” (P1_Home_3)  “This is my pussy cat […] I’m going to have it today [JfA cat]” (P1_Home_3)  “[JfA dog] Lassie, that’s a good name, you’re lovely aren’t you, you wouldn’t hurt me would you, you’re a good boy” (P1_Home_3)  “I’m going to miss him, miss him won’t I, they said, you’ll be alright, I want one, oh my darling, very lovely” (P2_Home_3)  “[strokes cat] I could do this all day” (P5_Home_3)  “How much for one? [Dog]” (P6_Home_3), “About 80” (Researcher), “Oh my gosh! Bit pricey” (P6_Home_3)  “Don’t you take mine [dog]” (P5_Home_1)  “Sold, I would like that [hedgehog]” (P11_Home_1)  “I’d like to take him home [JfA dog]” (P1_Home_2)  “What are you called, Snowy? [Paro]” (P1_Home_2)  “What are you called, Chatterbox? [JfA dog]” (P1_Home_2)  “This is mine [holds up cat]” (P1_Home_4)  “I’ll call him fluffball” (P2_Home_5)  “Are you Ginger, or do we call you Ginge?” (P5_Home_5)  “Is you name Dillon, is it? Or do they call you Cowboy [JfA dog]” (P5_Home_5)  “I’d like you in my bed! [Paro]” (P8_Home_5) |
|  | **Interest in Technology** | “Are they electric?” (P2_Home_3)  “What is the energy source?” (P2_Home_4)  “How does it work?” (P2_Home_2)  “They are robots” (P4_Home_3)  “Oh I see, the more you stroke them it starts off their repertoire” (P2_Home_4)  “They’re so well made” (P2_Home_4)  “Who made these then?” (P1_Home_5)  “I’d like to see what’s on the inside of them” (P5_Home_5)  “Tremendously well done [Miro]” (P1_Home_5)  “Look at this one, electric, this one’s electric [Paro]” (P4_Home_3)  “They aren’t real are they, robots!” (P6_Home_3) |
| **Focal Point** | **Conversation** | P6_Home_1: You wouldn’t bite me would you?  P5_Home_1: He wouldn't.  P6_Home_1: You would! (to dog) You would bite me?  P5_Home_1: He said, “Try me and see.” [Laughs]  P6_Home_1: You would bite me would you? Hey?  P5_Home_1: Do you know what it is?  P6_Home_1: I don't actually. Do you?  P5_Home_1: A seal.  P6_Home_1: I thought they lived in the water.  P2_Home_4: Oh you’ve got the seal, I used to see the off the Scottish coast  P1_Home_4: Yes, I can feel him rubbing into my side, oh yeah I’ve seen lots of these  P1_Home_5: Mind my cat!  P2_Home_5: It’s a dog darling [laughs]  P1_Home_5: [laughs] I do need to see the optician don’t I!  P6_Home_5: How much is that doggy in the window  P7_Home_5: The one with the waggily tail  P6_Home_5: How much is that doggy in the window  P8_Home_5: I do hope that doggies for sale  P6_Home_5: He’s laughing at you [Furby]  P8_Home_5: He’s laughing because I’m tickling his belly  P6_Home_5: Oh I thought he was laughing at your face! [laughs]  P8_Home_5: [Laughs] he might be! |
