# Supplementary material for: User-Centered Design of Companion Robot Pets Involving Care Home Resident-Robot Interactions and Focus Groups With Residents, Staff, and Family: Qualitative Study
Source: JMIR Rehabil Assist Technol. 2021 Nov 1;8(4):e30337. doi: 10.2196/30337 (PMC8593804; doi:10.2196/30337)
Supplement: Multimedia Appendix 2 [file rehab_v8i4e30337_app2.docx]

**Multimedia Appendix 2.** Further evidence from focus groups.

| Question  *Codes generated* | Example evidence |
| --- | --- |
| Favourite?  *Codes: domestic pet preferred, good acceptability* | P2: Cat (Home_2)  P1: Breathing dog (Home_2)  P2: Cat (HOME_1)  P1: Dog, or cat (HOME_1)  P3 (HOME_1): Hedgehog, cat, dog  P4 (HOME_1): cat  P5 (HOME_1): dog  P6 (HOME_1): “the cat is yeah”  P7 (HOME_1): dog “this one here”  P8 (HOME_1): “I think that little dog (joy)”  P1 (Home_3): “I think I’ll have a pussy cat, we got our precious moments”  P4 (Home_3): “Yeah the dog”  P2 (Home_3): I don’t honestly know, I don’t mind that [cat], beautiful, my darling, treasure pet”  P7 (Home_3): “I’d rather have that one” (Cat)  P6 (Home_3): “The cat”  P5 (Home_3): “I think the big white one”  S2 (HOME_1): “The cat, definitely the cat”  S1 (HOME_1): “The sit up dog”  F1 (HOME_1): “The kitten”  F3 (HOME_1): “The cat yeah”  F2 (HOME_1): “The one that turned over and meowed and purred”  S1 (Home_2): The cat the dog and sleeping dog  S2 (Home_2): “The seal, the dinosaur and the dog”  S3 (Home_2): “Yeah same [seal, dinosaur, dog]”  F1 (Home_2): “I preferred the seal, I like seals, very soft and strokable”  F2 (Home_2): “I actually liked the Furby because I thought it was quite amusing”  S1 (Home_3): “the cat, I think, most popular, we’re never gonna get a seal in here”  S2 (Home_3): “The cat”  S3 (Home_4): “I think the cat is pretty amazing  F1 (Home_4): “That’s the best [dog]”  S4 (Home_4): “The dinosaur”  S5 (Home_4): “I think the cat”  S6 (Home_4): “I would say dinosaur”  S7 (Home_4): “The seal, it’s amazing, I want one”  S8 (Home_4): “I can see the appeal of the Furby, the seals lovely”  F2 (Home_4): “The cat, actually I’m going to put the cat in my bag”  S9 (Home_4): “I like the dog in the basket over there, I like that [Perfect petz]”  P1 (Home_4): “The best is the cat”  S10 (Home_4): “The cat is amazing”  F1 (Home_4): “The puppy”  P2 (Home_4): “The dinosaur”  S11 (Home_4): “The cat, or dog”  S12 (Home_4): “The cat”  S13 (Home_4): “I like the cat and the dog”  F3 (Home_4): “Oh I like this one [Paro] but she likes the cat the most”  F4 (Home_4): “The seal is the best one”  P5 (Home_5): “My favourite would be the cat”  P1 (Home_5): “I think the seal”  P6 (Home_5): “Furby”  P7 (Home_5): “I don’t really like them”  S4 (Home_5): “The big dog, with the neck tie”  S3 (Home_5): “Yeah I prefer the dog as well”  S6 (Home_5): “I like the cat”  P1 (Home_5): “I can’t say that I have a particular favourite because they’re so good all of them”  P3 (Home_5): “Him [Furby]”  P2 (Home_5): “For children”  P4 (Home_5): “The dog in the basket [Perfect petz], but now I’ve got him against the cat, the cat takes over”  P5 (Home_5): “Probably the cat”  P7 (Home_5): “I haven’t got a favourite, I like them all”  P8 (Home_5): “I like them all”  P6 (Home_5): “I like them all, I haven’t got a favourite”  S7 (Home_5): Seal  S8 (Home_5): Seal  S9 (Home_5): Cat  S10 (Home_5): “cat and [Perfect Petz] dog”  F1 (Home_5): “Cat” |
| Why  *Codes: Realistic embodiment, interactivity, domestic pets, soft fur, attractive, acceptability, negative response, weight and size* | P2 (Home_2): Realistic  P1 (Home_2): “So real”  P2 (HOME_1): “it was so much a cat” “so real” “seems so real” “Very realistic” “And the cats size as well, not like that seal” P3 (HOME_1): “It’s so real and so clever (hedge)” “very realistic” (cat) “it’s strong and it’s movements, it’s so real (dog)” “you’ve got all the mannerisms in the dog, it is a dog”  P5 (HOME_1): (dog) “the one I talk to, ain’t it mate, that’s right I agree with you (to dog)”  P5 (HOME_1): “because he talks to me and he tells me, he bloody talks to me, believe me, aint you, you’re watching and you’re listening, yes”  P4 (HOME_1): “You’d think he was a real cat, looking like that there, you’d think he was a real cat”  P6 (HOME_1): “well he’s so real looking”  P8 (HOME_1): “I just liked the way he moved”  P1 (Home_3): “Not too yappy”  P4 (Home_3): “Beautiful”  P2 (Home_3): “They’re beautiful”  P5 (Home_3): “I think they’re all lovely […] beautiful”  P7 (Home_3): “You can’t do anything else but love the cat”  P5 (Home_3): “Natural, but that one is a waste of time [Furby]”  S2 (HOME_1): “Everybody […] will stroke a cat or a dog, who strokes a seal? Nobody does do they”  S1 (HOME_1): “they, they were more interactive and made the noises like they should make and they were like looking at the residents.”  S2 (HOME_1): “They were nice and easy”  S2 (HOME_1): “They could sit on your lap comfortably”  F1 (HOME_1): “Absolutely brilliant. Yeah very tactile”  F2 (HOME_1): “They engaged the people that they are intended for as well, which I think is an important thing”  F2 (HOME_1): “She liked the one that turned over and meowed and purred I think”  S1 (Home_2): “I think they’re more realistic, they are more something that they would be used to. They’re not likely to have come across seals”  S2 (Home_2): “I think those three would be more interactive for the residents that we’ve got”  F1 (Home_2): “Very soft”  F2 (Home_2): “Amusing”  S1 (Home_3): “Looks more realistic, the cat looks most realistic and I think that’s what they respond to and the cat is more responsive […] it does more, they get more response from it”  S2 (Home_3): “She was like oh my beauty, she’s a cat lady”  S3 (Home_4): “Cat because it can sit on a lap and do thing”  S2 (Home_4): “For people without sight, or, that’s a really quite comforting feeling, that’s quite reassuring and really makes you feel calm I think”  S4 (Home_4): “I like it when he clings to you when you hold him up [Pleo]”  S5 (Home_4): “I think cats are comforting, and feeling the purring, for someone who couldn’t visualise or see it, you’ve got that kind of tactile”  S6 (Home_4): “[Pleo] I like the way he reacts to everything, he seems to do a lot, and he grips you when you pick him up”  S7 (Home_4): “It’s just gorgeous, lovely”  S8 (Home_4): “The weight on the seal if quite comforting, if someone’s agitated”  F2 (Home_4): “[Husband] always liked cats”  F1 (Home_4): “Well, he does everything he should do, he wags his tail, moves his head, he’s the right size, dog like that’s it yeah”  F2 (Home_4): “I think you had a dog yeah, do you see what I’m getting at, the association”  P1 (Home_4): “What I liked about the cat, I mean, a lot of things, his face was very good, eyes are, and the wrinkles in his ears, and the furs just fine”  S11 (Home_4): “Seems the best effect”  S12 (Home_4): “This is the lovely but the cost is too much, and they’re tactile as well, they make noises and”  S13 (Home_4): “Because that’s what the residents have had at home (cat or dog) and that’s what they’re used to, especially those that come in and can’t bring their dog with them”  F3 (Home_4): “Very realistic, the feel and what it does”  F4 (Home_4): “Just so realistic with what is does, and the eyes and the fur”  P2 (Home_5): “[Paro] because it’s so cuddly”  P6 (Home_5): “[Furby] it just looks nice”  P2 (Home_5): “The eyes are so animated [Furby]”  P5 (Home_5): “Because I just love cats”  P4 (Home_5): “At the risk of being thought stupid, because it looks like a cat!”  P7 (Home_5): “They’re just acting like they’re real, yeah the movement I think”  P6 (Home_5): “I like the feel of them”  S7 (Home_5): “I just like cats, and how it is realistic like”  S9 (Home_5): “How it [Paro] feels”  S10 (Home_5): “They’re the right shape, the right size, the right texture, they’ve had cats and dogs, but that dog is too raring to go [JfA] might be a bit intimidating”  F1 (Home_5): “We are both cat people, it would be comforting and calming and the texture is important, when you’re losing a lot of senses, the ones you have, if they can be stimulated it’s really important” |
| New robot design?  *Codes: Realistic embodiment, domestic pets, life simulation, command response, weight and size, practicalities,* | P1 (Home_2): “Already got my favourite!  P2 (Home_2): “A cat maybe”  P2 (HOME_1): “a little bit of thought to the cat to start with – I’ve never seen a cat that colour” P2 (HOME_1) realistic? “Yes, oh absolutely”  P1: “I like the way they done this one at the front and made him very realistic” “I like the green eyes”  P3 (HOME_1): “the cats fur is a bit too long”  P3 (HOME_1): “the dog should sit up and beg”  P4 (HOME_1): “have it walk” “go round your legs”  P1 (Home_3): “No sounds, wakes somebody up”  S1 (HOME_1): “Quite robust as well”  S1 (HOME_1): “They will probably need to be washable to a point”  S2 (HOME_1): “Covers come off”  F3 (HOME_1): “When they have their snooze and they drop off, it drops off and doesn’t disturb them”  F3 (HOME_1): “The fabric, what, can you take it off and wash it? Because you know, they’re coming away after dinner, they forget because they’re old and, it gets greasy and mucky”  F2 (HOME_1): “The odd bit of apple crumble you know. I could see it getting quite dirty after a while”  S1 (Home_2): “I think breathing Is good, the noise, whatever animal it is, it makes and then a little bit of movement, I like the cat moving his paw and things like that”  S2 (Home_2): “Maybe softer, softer feeling I think. In the body. So robotic. If you added something a bit softer it will feel more, like that they’re touching real animals really.”  F1 (Home_2): “I think the cat or the dog, I found it wasn’t as soft as the seal, so maybe it didn’t feel, it felt a bit more kind of”  F1 (Home_2): “I guess wanting to play like a dog, engage in some kind of play […] and wanted it’s tummy rubbed and things like that […] being able to feed it something”  S1 (Home_3): “I think interactive is good, because I think they get more out of it, and I think it has to be reasonable weight, I think weight is important, […] some older people are quite frail, so they could sit with the cat, who’s still quite heavy but more accessible than if you put Paro on them because it might be too heavy and then they’re not going to interact with it as much”  S1 (Home_3): “The right shape to go on their lap, the cat, is perfect to go on a lap, you can just have it there but the dog it too upright, too rigid”  S6 (Home_4): “It needs to be washable”  S5 (Home_4): “Hm yes it needs to be washable and not white”  S8 (Home_4): “The weight of an animal”  F1 (Home_4): “It could be a bit lighter, that’s only my opinion, and not too much like a toy, they could take offence, it’s like when they try and give them children’s puzzles, they’re adults”  P2 (Home_4): “I think repetitive friendship, not too many activities”  F4 (Home_4): “Quite realistic looking, not too much, way they move, could do rabbits”  S4 (Home_5): “I just think they need to be more realistic pets, with the fur, and animal noises”  P2 (Home_5): “You want a Labrador that opens the fridge and gets you a beer”  P5 (Home_5): “What about a fish. I’d still go for something like the cat”  P4 (Home_5): “Well it was lovely to see the cat moving. It’s gotta meow hasn’t it”  S9 (Home_5): “Realistic”  S10 (Home_5): “Look like something they had in the past or it will be alien to them, stick to cat or dog, they’re not into hamsters or chinchillas, something for their lap, help calm them down and relax them”  F1 (Home_5): “Soft is appealing, the response, it’s nice when the cat will meow, turns its head towards you, loved it turning over for tummy to be tickled”  F1 (Home_5): “Something warm, purring on her lap” |
| What would you like it to do?  *Codes: Interactivity, command response, life simulation,* | P1 and P2 (Home_2) “no” to being non-interactive  P2 (HOME_1): interactive? “Yes that’s the idea of a robot”  P2 (HOME_1): “he turned over just now” “well that was good”  P3 (HOME_1): “all that is very clever, they all do that kind of thing, cats don’t do much”  P5 (HOME_1): he talks at me and he looks at me  P11 (HOME_1): I think the one that’s breathing [sic] you want it to play, a bit more action”  P4 (Home_3): “Teach them not to bite anybody because they’ll get into trouble”  S1 (HOME_1): “[Paro] probably too complex really for their needs”  S1 (HOME_1): “It would be nice if it could say […] roll over or beg”  F3 (HOME_1): “It should have sound […] you want to touch it, you want to hear it and you want to see it move”  F2 (HOME_1): “I think movement is actually a good thing [because if you then tell it to stop moving or sit or something it gives them vocabulary that they might have forgotten”  F3 (HOME_1): “But to have something that is there and the cat is going to turn over and still keep breathing”  F1 (HOME_1): “And to be able to feel the purring”  S3 (Home_2): “They like the noises in the background. Because they like interacting”  S2 (Home_2): “It got to be interactive […] so residents have something to have their minds think about as well”  S2 (Home_2): “The lights on the dog, I liked. I think that was pretty cool”  F1 (Home_2): “Kind of like temperature, like warmth”  S1 (Home_3): “I think they’re looking for responsiveness to hold their attention span, […] oh it’s looking at me or oh it’s vibrating or purring”  S1 (Home_3): “For me, it’s that looking for them, I’m not sure how many times they’d notice the tail wagging because they’re probably looking at its face […] the heads moving, eyes opening and closing”  S6 (Home_4): “It has to be interactive, that [hedgehog] is fun but in time you’d lose interest”  F2 (Home_4): “I don’t think it needs to be any bigger than this really [JfA dog], lap size, because most people spend their time sitting down”  S13 (Home_4): “Movement, looking at me”  S12 (Home_4): “interactive to the person”  S10 (Home_5): “Adaptable to the person, something like that [Perfect petz] that is so peaceful and relaxing to look at, but it can do other things when needed, if you’re gonna make something make it wide ranging, make it as adaptable as possible”  S10 (Home_5): “Size and weight, this [Paro] is far too bulky, so big and heavy for the older ladies”  F1 (Home_5): “Respond to her” |
| Feel?  *Codes: Soft fur, tactile, dislike plastic* | P1 (Home_2): “Soft I think”  P2 (Home_2): “Yeah”  P2 (HOME_1): “that one” (soft) “I’ve always had a cat you see”  P3 (HOME_1): “rough haired and smooth haired” “it’s a question of choice” “I don’t like the plastic ones”  P4: “the furry ones I think” “that looks a lot better… you’d look at it and think it was a real one”  P6 (HOME_1): definitely soft ones  P12 (HOME_1): Just like the plastic one  P11 (HOME_1): “I don’t like the plastic ones”  P6 (Home_3): “[dinosaur] that’s very unusual feel”  P6 (Home_3): “[Pleo] feels quite hard”  P7 (Home_3): “Oh the soft ones”  P5 (Home_3): “I think it’s gorgeous.” (cat)  P5 (Home_3): “That one I think is very natural”  P5 (Home_3): “This one is lovely because of it’s hair”  S2 (HOME_1): “they were pleasant to touch, the furry ones”  S1 (HOME_1): “Soft”  S1 (HOME_1): “The plastic ones aren’t quite so. Sort of sit on your lap sort of thing”  S2 (HOME_1): “Soft to the touch and they could stroke it”  S1 (Home_2): “Soft”  S3 (Home_2): “Really fluffy”  S2 (Home_2): “The dinosaur quite good. That’s got quite a soft but hard texture. And the seal and the dog and the cat, they are quite soft. Really fluffy, so something like that I think would be more. It would be soft and fluffy”  F2 (Home_2): “I think furry myself”  F2 (Home_2): “I think actually stroking something soft if quite beneficial”  S1 (Home_3): “Soft”  S6 (Home_4): “Fur, I think so”  F1 (Home_4): “I like the fur”  P2 (Home_4): “The dog has got the best fur”  P1 (Home_4): “The fur is fine”  S10 (Home_4): “The fur is more tactile”  F2 (Home_4): “I think it needs to be something furry like this”  S13 (Home_4): “The hair, tactile ones”  F3 (Home_4): “The fur”  F4 (Home_4): “Furry ones probably best”  S4 (Home_5): “Fur, I would think so”  S3 (Home_5): “Yes fur”  P6 (Home_5): “Yeah fur”  S5 (Home_5): “I don’t like the things that look like toys, I prefer the furry things”  S1 (Home_5): “The fur is more therapeutic”  P2 (Home_5): “I wouldn’t like plastic, it would be too cold”  P4 (Home_5): “Furry”  P5 (Home_5): “I prefer the furry, personally”  S1 (Home_5): “For residents, the fur”  P8 (Home_5): “I like the furry ones”  P6 (Home_5): “No I don’t like the rubber ones, the furry ones look more real, you don’t get rubber animals, you get furry ones”  P7 (Home_5): “The rubber one interacted anyway so I’ve got no preference”  S7 (Home_5): “Soft fur”  S8 (Home_5): “Soft furry stuff”  S9 (Home_5): “Soft fur”  S10 (Home_5) “Furry”  F1 (Home_5): “I think […] it needs to be something that can be stroked, soft, it’s nearer to the animal, the texture is comforting” |
| Expressions? Behaviours?  *Codes: Facial expressions, gaze direction, life simulation, interactive* | P1 (Home_2): “Mouth moving” “walking”  P2 (HOME_1): “that one” (cat) “It’s got an expression and it looks at you”  P5 (HOME_1): “Ones that talk to you” “I like the ones that talk to you”  P4 (HOME_1): “I was going to say [sic] seeing how he turns his head”  P5 (Home_3): “They’ve all got their own thing about them”  S1 (HOME_1): “The one sat in the basket was just heart beating and no one was particularly that interested”  S1 (HOME_1): “Head movements as well, the way it’s like looking at them or looking then looking back at them”  F1 (HOME_1): “Give me a paw”  F1 (HOME_1): “Probably wagging the tail for the dog, because that’s like, the dogs excited to see you type of thing isn’t it? I suppose cat purring you would get that, but then cats waggle their tails too don’t they?”  S1 (Home_2): “Do the cats eyebrows move and eyelids move or anything? Because that would be quite good”  S2 (Home_2): “Open his mouth, move his eyes, wag his tail. Maybe some sounds, like when it’s sad or happy. Yes, some moods I think. […] they all have different mood swings sometimes, so it would be nice to have something where, if they are feeling sad we can say, right well here you do […] lights up to show their moods, so we could set it on, so that is will make them more chilled, or happy, placid mood”  F1 (Home_2): “Something that’s a bit playful and happy, maybe a bit comical”  F2 (Home_2): “Facial, I think because it’s the first sort of thing”  F1 (Home_2): “Yeah I think facial but I think more body as well, so if yes, with the dog, you can have rolling over or wagging tail”  S2 (Home_3): “Breathing, once she realised it was breathing, she was like aw, she wanted to listen”  F1 (Home_4): “The mouth, opening, closing”  S5 (Home_4): “The looking, the looking, that sort of interaction, and I mean the tail, the rolling over to have its tummy tickled”  P2 (Home_4): “Older people are not so interested in the flexibility and that sort of thing, only something you come home to, and have every day and you know where you are in the world”  P1 (Home_4): “The eyes, the eyes”  P2 (Home_4): “Faces are supposed to be what people look at, see the eyes moving”  S10 (Home_4): “It’s not just the head movement, it’s all of the features, makes it so realistic for”  S12 (Home_4): “To look towards you”  F3 (Home_4): “Realistic animation, but softer fur, similar to Paro, the cat feels quite course, normally smooth”  F3 (Home_4): “React like a normal animal would, like the cat is”  F4 (Home_4): “When it opens its eyes, it’s like it’s talking to you, the Furby is a child thing”  S5 (Home_5): “The breathing is relaxing”  S4 (Home_5): “I like the animated eyes”  P6 (Home_5): “I like the eyes”  P2 (Home_5): “I think so, that’s very amusing”  P3 (Home_5): “Nice to have the animation, I think the animation makes everyone engaged”  P2 (Home_5): “The more it does the more interesting it is”  P7 (Home_5): “Yes they’ve got to, and their movement that’s what makes them look real”  P6 (Home_5): “I like them purring”  P8 (Home_5): “I love to hear them purr”  S7 (Home_5): “Interactive is better”  S8 (Home_5): “Blinking, wagging tail, moving, purring, even when stroking you can feel the cats purring even if they can’t hear it”  S10 (Home_5): “Rolling over is lovely” |
| Features to avoid?  *Codes: Dislike plastic, weight and size, non-interactive, appropriate noise, appropriate movements* | P3 (HOME_1): “I think that one which breaths. It looks as though it’s breathing, you want it to play” “it could do with being a bit more”  P4 (HOME_1): “I like them all”  S2 (HOME_1): “Paro tended to be a bit big and heavy”  S1 (HOME_1): “Plastic”  S2 (HOME_1): “Moving it around the floor”  F3 (HOME_1): “The dog […] nice but it just sits there”  F2 (HOME_1): “[Dog] it could do a bit more”  F3 (HOME_1): “[Dog] it could do with being a bit more mobile”  F2 (HOME_1): “Anything plasticy”  F2 (HOME_1): “That is unreal”  F3 (HOME_1): “The dinosaur looked like a plastic toy, it wasn’t particularly attractive. It felt like gooey rubber”  F2 (HOME_1): “You wouldn’t want to pick it up and cuddle it, would you? [Pleo]”  S2 (Home_2): “I think they would get bored with that one [Perfect petz]”  S3 (Home_2): “I think they’re going to get bored with that [Hedgehog]”  F1 (Home_2): “I don’t think lights”  F2 (Home_2): “Something that doesn’t do anything”  F1 (Home_2): “Hard shell or lights”  S1 (Home_3): “The Furby has been very unpopular”  S1 (Home_3): “Anything running around the floor is a trip hazard, you’ve got people that are unstable, […] they can’t always see”  S1 (Home_3): “The movements can’t be too fast, you’ve got people with sight impairments, cognitive impairments, they can’t process it quick enough [Miro]”  S1 (Home_3): “Not, not touchy feely, you haven’t got that sensory feel like you’ve got with the animals”  S1 (Home_3): “A quiet little purr is fine, but you don’t want a dog barking away, but maybe a volume as if someone was hard of hearing or visually impaired they might need that to make it more responsive”  S5 (Home_4): “Movements too quick”  F1 (Home_4): “Too loud”  S6 (Home_4): “I think we don’t want any hazards, you know it could trip you up if it moved too quickly”  P2 (Home_4): “The barking would irritate the other residents, not talking all the time”  S13 (Home_4): “Avoid something like that, hard [Miro] this is nicer for them [soft]”  F4 (Home_4): “Spiders, snakes, I think well”  S1 (Home_5): “I’m not keen on these because they’re like toys [Pleo and Miro]”  P1 (Home_5): “I’m not keen on those two [Pleo and Miro] because I’m used to animals”  S2 (Home_5): “Plastic”  S3 (Home_5): “Plastic”  P5 (Home_5): “I think this is like a childlike thing really [Miro] kids would like that”  P7 (Home_5): “Plastic no”  P6 (Home_5): “No rubber ones, don’t get a rubber animal anyway”  S6 (Home_5): “Plastic”  S7 (Home_5): “Take itself for a walk around the building”  S8 (Home_5): “Rubber”  S8 (Home_5): “Too fast, too erratic, too loud”  S10 (Home_5): “Not too heavy, no ants, insects, spiders”  F1 (Home_5): “Plastic, it would be alien to what she would expect” |
| Talking?  *Codes: Animals noises, negative response, positive response, unrealistic,* | P1 (Home_2): “they all talk like you, we wouldn’t get a word in edge ways”  P1 (Home_2): “Well can’t understand an animal language can you?”  P1 (Home_2): “We get to know what they want, you know, movements and that”  P2 (HOME_1): I don’t think I would go with speech, that’s a bit fanciful, I think, I just like the… just the animal noises”  P3 (Home_3): “I like both (talking or animal noise) anything you do with it, they’re lovely” P4 (Home_3): “I don’t think it matters” “I like to listen to the English being spoken, there’s times when I think, of well, I’d rather listen they talk to me than me just listening there to them [sic] but as I say, don’t matter to me whether they”  P6 (HOME_1): I would think that’s wonderful, wouldn’t you? I wouldn’t want them answering back though” “because I’d get cross in the end I expect”  P5: “I’d say you were nuts and I was nuts” “round the bend good and proper”  P7 (HOME_1) “ wouldn’t do any good that”  P1 (Home_3): “They can’t talk to us, I’d like it if he spoke back”  P4 (Home_3): “I think you’d get tired of them”  P5 (Home_3): “No, don’t know”  P7 (Home_3): “I think you’re always aware that they are what they are, that’s the trouble” “So they’re better barking and meowing?” “Yes”  S1 (HOME_1): “I think the animal, the noise of the animal. I don’t think they want to get into conversations with it. Just a bit of companionship”  F1 (HOME_1): “I think it should just make animal noises”  F3 (HOME_1): “How much is a dementia patient going to understand? […] So an animal speaking to them is no different to a human being”  F2 (HOME_1): “The only thing with Furby’s, you can talk to them and then they will repeat what you said, can’t you? So I suppose somebody whose language is going that could encourage them to speak”  F1 (HOME_1): “Maybe for speech therapy yeah. You could link that with speech therapy […] and then that would work for stroke victims as well”  S2 (Home_2): “I think that would be ideal. I think so. Because they might be able to express their feelings more than what they can do to a carer or to a doctor. They, you know, they might be able to express more if it’s something”  S3 (Home_2): “Yes”  F1 (Home_2): “I don’t know whether it will be good to have that’s like a real animal talking, whether they’ll think that’s just too weird or”  S1 (Home_3): “I don’t think that’s going to be good necessarily, because it’s an animal making the noise, so processing that information might be a sensory overload, like processing why is a cat talking to me”  S5 (Home_4): “I think probably the animal noises, to make it more realistic”  S6 (Home_4): “Yes I agree, I think it could be a bit disturbing having a human voice coming out of an animal”  them”  P1 (Home_4): “No, the sound of the animal”  P2 (Home_4): “No, not worth the effort”  S12 (Home_4): “No I don’t think so no”  S13 (Home_4): “No, animal noises”  F3 (Home_4): “Stay with animal noises”  F4 (Home_4): “For people who do talk, yeah, some people their language is a bit, could be a good idea”  S1 (Home_5): “I don’t think that’s necessary”  P2 (Home_5): “Yes if they could interact with you, hold a conversation, that would be very interesting”  P3 (Home_5): “Yes would be interesting, as long as it had an off button as well”  P5 (Home_5): “Talk, yeah, he could [Furby] but don’t talk my word for it”  P4 (Home_5): “I’d rather they made animal noises”  P7 (Home_5): “Yeah I think it’s great, but if they want to bark let them bark, but he’s great [Furby]”  S8 (Home_5): “Not talking, a lot of them are deaf and don’t understand a lot of the time anyway”  S10 (Home_5): “Language would be difficult, deafness is a huge problem, but even if hearing isn’t that bad it needs to be slow and clear, it gets lost, they wouldn’t expect an animal to talk”  F1 (Home_5): “I don’t know, it is, I’d be quite happy if it conversed with me, but it’s possible a furry creature talking back to mother might freak her out, again, her hearing is going” |
| Personalisation?  *Codes: Positive response, not necessary, past pet, attachment,* | P1 (Home_2): “Sounds alright” “I knit, I knit everything” “Yeah why not”  P2 (Home_2): “I knit yeah” P2 (HOME_1): “That’s okay if people want to do that yes” “you can choose your own colour then.”  P6 (HOME_1): “How I like, which animal I’d like, that’s nice”  P11 (HOME_1): “I think they’re done well enough aren’t they”  P11 (HOME_1): “A teddy bear would be good doing that […] yes a rabbit, I like rabbits”  P7 (Home_3): “Well I suppose I would personally”  P5 (Home_3): “No I think they’re all lovely”  S1 (HOME_1): “It would be nice if they could choose. But is that robot going to be personable to them or is it just going to be a robot in a home, everyone’s going to have different opinions”  S1 (HOME_1): “it would be nice if they could choose their particular features what they would like their cat to look like or dog or”  S1 (Home_2): “I suppose they could have it so it looks like a pet that they’ve had in the past. But then you’ve also got the thing of, when that persons gone, that animal is not going to be significant for anyone else, so cost wise, it wouldn’t be cost effective, unless you could change the outer”  S3 (Home_2): “That would be good because we have quite a few here that like knitting.”  S2 (Home_2): “I think a couple of them can actually knit and crochet, so that would be quite a nice idea. They can knit their own companion, or for the others that don’t, obviously they can tell their ideas to someone and hopefully they could make. I think so, it would feel like they’re then part of something then.”  S3 (Home_2): “They will just get more attached.”  F1 (Home_2): “I think it’s a really good idea”  F2 (Home_2): “I think it would give them a purpose, because, you go in most of them are sleeping every time […] you know they need something”  F1 (Home_2): “Yes it’s cool. They’ve put something into it and make it, reflect their personality and more wanting to engage and look after it I think”  S1 (Home_3): “I think that would be good for some, like [resident] who had a ginger cat, but for some people it might be upsetting, […] but for others it might be more comforting”  S2 (Home_3): “It’s like [resident] and her dog [teddy], that is her dog, but then with this one she said oh it’s electric”  S1 (Home_3): “It’s almost like you need a robotic framework that goes into every animal, and then the shell you could change”  S1 (Home_3): “A rabbit could be another idea”  S5 (Home_4): “That would be brilliant, I think, if they’ve had a cat, or whatever”  F1 (Home_4): “Yes, yeah”  P2 (Home_4): “If they were able to”  S12 (Home_4): “Good yeah”  S13 (Home_4): “Yeah I think that’s a good idea”  F3 (Home_4): “[Resident] would always prefer a black cat, if it was an option it would be good”  F4 (Home_4): “Yeah that would be good, Mum and Dad used to have a spaniel, red setter colour, something like that would be good for them, memory not great but might spark something off”  S4 (Home_5): “Having an input on the colour and things yeah”  S5 (Home_5): “There will be some residents that would like to be involved, more hands on”  P2 (Home_5): “Good yes”  P3 (Home_5): “Good if you can pull it off”  P5 (Home_5): “People could even gift it, yeah I agree with that”  P4 (Home_5): “Yes, definitely”  S1 (Home_5): “Yeah, I would like a grey coloured cat”  P5 (Home_5): “I would like a black cat, although this one is beginning to weasel in”  S1 (Home_5): “People could make covers that were washable and removable and be involved in the making of it”  P7 (Home_5): “Yeah I suppose it does”  S7 (Home_5): “I think that’s a good idea, they’ll have a connection with it, they’ll enjoy it”  S6 (Home_5): “They’ve made it themselves”  S8 (Home_5): “If they have dementia and live in a certain time period, they could create an animal from their time”  S10 (Home_5): “I think it’s absolutely brilliant if people can and would want to do that”  F1 (Home_5): “I think that’s a lovely idea, colour is important, if they’ve had an animal in their past that means something more to them” |
| Prefer realistic or unrealistic?  *Codes: Realistic, domestic pet, incongruence of unfamiliar,* | P1: “Real one”  P2: “Don’t know, that’s a knitted one?”  S1 (HOME_1): “I think real”  S2 (HOME_1): “Real […] it sort of stimulated their memories”  F1 (HOME_1): “I think it just goes back to a normal, domestic, animal or pet that you would have”  F3 (HOME_1): “It does all the things that you would hope you domestic cat would do probably”  F1 (HOME_1): “It’s easy to identify with the cat, whereas the seal, I think, well, why have you got a seal in a home? You wouldn’t necessarily have one”  S1 (Home_2): “It’s too futureristic [Miro], I think it needs to be more realistic”  S1 (Home_2): “A cat and a dog, with this generation”  F2 (Home_2): “I think the domestic animal, […] I don’t know whether the seal would go down as well”  F1 (Home_2): “The dog or the cat yes”  F1 (Home_2): “I would have thought they would prefer something that’s a bit more realistic, although I thoroughly like the dinosaur”  S1 (Home_3): “The dinosaur, because it’s not reality, people just see it as a bit of fun as opposed to a companion”  S1 (Home_3): “If it’s realistic they can relate to it more than Paro”  S2 (Home_3): “Yeah he thought the cat was just lovely, he used to have a cat very similar”  F1 (Home_4): “Realistic, I think”  S5 (Home_4): “Realistic yes”  P2 (Home_4): “People have more knowledge of cats and dogs”  S11 (Home_4): “I think most people would go for a cat and a dog because they’re used to it”  P1 (Home_4): “I don’t like the unrealistic ones”  S12 (Home_4): “Yeah I would have thought so [realistic]”  S13 (Home_4): “Yeah definitely [realistic], more realistic than futureristic”  F3 (Home_4): “The more realistic the better, a cat or a dog, the dinosaur is good as a novelty, it’s more therapeutic if they recognise it”  F4 (Home_4): “As realistic as possible really, a Furby would appeal to children more”  S1 (Home_5): “These are more like the real thing because they’ve got fur [JfA]”  P2 (Home_5): “I think I like something I recognise, for me”  S2 (Home_5): “That’s more realistic [cat] that’s important”  S3 (Home_5): “Yes I think so too”  P2 (Home_5): “Unrealistic is interesting, it would hold your gaze because it’s different”  P3 (Home_5): “It raises expectations if it’s realistic doesn’t it”  S1 (Home_5): “Replicating a real animal that may even bring back memories of their own animals”  P4 (Home_5): “Lifelike”  P5 (S): “I think the real animal myself”  P7 (Home_5): “I don’t mind, he’s making me laugh [Furby]”  P8 (Home_5): “I like them realistic”  S6 (Home_5): “These are better for people with learning disabilities [Miro and Pleo]”  S6 (Home_5): “Realistic”  S7 (Home_5): “Yeah [realistic]”  S8 (Home_5): “Yep [realistic]”  S10 (Home_5): “Very, very realistic, I think it should be”  F1 (Home_5): “It could be something less recognisable, although, perhaps, some of the residents would take against something they perceive as a toy” |
| Keep one?  *Codes: Domestic pets preferred, good acceptability* | P1 (Home_2): “The dog” (breathing)  P2 (Home_2): “The cat”  P2 (HOME_1): “the cat”  P3 (HOME_1): “the hedgehog”  P5 (HOME_1): “naturally it would be you (d0g)”  P6 (HOME_1) “I like the cat”  P7 (HOME_1) “I would say this one” (cat)  P11 (HOME_1): “I wouldn’t want one”  P19 (HOME_1): Cat  P1 (Home_3): Cat  P4 (Home_3): “Cat, I enjoyed it up to a point”  P2 (Home_3): “Dog, yes it’s a love”  P7 (Home_3): “I would go for the cat”  P5 (Home_3): “A pretty looking cat”  S2 (HOME_1): “I think the dog or the cat”  S1 (HOME_1): “Yeah it would be the dog or the cat”  S1 (Home_2): “I think the cat and maybe the dog in the basket”  S2 (Home_2): “The dog, the dinosaur and the seal”  S3 (Home_2) Dog, dinosaur and seal  F1 (Home_2): “The dog or cat”  F1 (Home_3): “I’d keep the cat and the seal”  S5 (Home_4): “Cat”  S6 (Home_4): “Dinosaur”  S11 (Home_4): “The one that [resident] likes [cat]”  P2 (Home_4): “The little hedgehog”  P1 (Home_4): “Cat”  S12 (Home_4): “Yeah, the cat or the dog”  S13 (Home_4): “The cat or dog, out of everything”  F3 (Home_4): “For me the seal, for [resident] the cat, to be honest for the reactions, I’d pick the cat”  F4 (Home_4): “The seal actually”  S6 (Home_5): “Yes I’m taking this one [cat]”  S4 (Home_5): “I’d probably have the cat”  S5 (Home_5): “Yes I’d have the cat”  P6 (Home_5): “Cat”  P7 (Home_5): “No”  P2 (3): “Furby”  P1 (Home_5): “Furby is as good as any”  P3 (Home_5): “He’s the one [Furby]”  P4 (Home_5): “Cat”  P5 (Home_5): “It would be the cat, I’ve got to admit that”  S1 (Home_5): “That one [Cat]”  P7 (Home_5): “I’d pick the lot, but the dog I think”  P8 (Home_5): “I’d keep them all”  S7 (Home_5): “Cat or seal”  S6 (Home_5): “Cat or seal, dog possibly”  S10 (Home_5): “Cat and Perfect Petz dog”  F1 (Home_5): “Cat” |
| Technology experience | P1 (Home_2): None  P2 (Home_2): None  P2 (HOME_1): iPad  P1 (HOME_1): No  P3 (HOME_1): No  P5 (HOME_1): No  P2 (Home_4): “I did use a tablet”  P2 (Home_5): No  P1 (Home_5): No  P5 (Home_5): “no”  P4 (Home_5): No  P6, p7, p8, no |
| Pets | P1 (Home_2): “Yeah, always had a dog” “And we’ve had a cat sometimes”  P2 (Home_2): “Always had a dog”  P3 (HOME_1): “oh, cat, cat or a dog, I always had a dog”  P2 (HOME_1): “yes cat”  P5 (HOME_1) dog  P8 (HOME_1): “I was a dog man”  P10 (HOME_1): cats and dogs  P2 (Home_4): “We always had a cat” |
| Ages | 84, 92, 96, 101, 98, 93, 92, 88, 90, 70, 89, 78, 86, 87, 91, 97, 77, 90, 91, 99, 75, 89, 95, 92, 91, 96, 92, 86 |
